# Bat responses to changes in forest composition and prey abundance depend on landscape matrix and stand structure

**DOI:** 10.1038/s41598-021-89660-z

**Published:** 2021-05-19

**Authors:** Jérémy S. P. Froidevaux, Luc Barbaro, Olivier Vinet, Laurent Larrieu, Yves Bas, Jérôme Molina, François Calatayud, Antoine Brin

**Affiliations:** 1 Université de Toulouse, INRAE, UMR DYNAFOR, Castanet-Tolosan, France; 2 University of Bristol, School of Biological Sciences, Life Sciences Building, Bristol, UK; 3 CESCO, Museum National D’Histoire Naturelle, CNRS, Sorbonne-Université, Paris, France; 4 Office National Des Forêts (ONF), Agence Etudes Midi-Méditerranée, Montpellier, France; 5 CRPF-Occitanie, Antenne de Tarbes, Tarbes, France; 6 Centre D’Ecologie Fonctionnelle Et Evolutive (CEFE), Université de Montpellier, CNRS, EPHE, IRD, Université Paul Valéry Montpellier 3, Montpellier, France; 7 Université de Toulouse, Ecole d’Ingénieurs de PURPAN, UMR INRAE-INPT DYNAFOR, Toulouse, France; 8 Present Address: Biological and Environmental Sciences, Faculty of Natural Sciences, University of Stirling, Stirling, FK9 4LA UK

**Keywords:** Biodiversity, Forest ecology

## Abstract

Despite the key importance of the landscape matrix for bats, we still not fully understand how the effect of forest composition interacts at combined stand and landscape scales to shape bat communities. In addition, we lack detailed knowledge on the effects of local habitat structure on bat-prey relationships in forested landscapes. We tested the assumptions that (i) forest composition has interacting effects on bats between stand and landscape scales; and (ii) stand structure mediates prey abundance effects on bat activity. Our results indicated that in conifer-dominated landscapes (> 80% of coniferous forests) bat activity was higher in stands with a higher proportion of deciduous trees while bats were less active in stands with a higher proportion of deciduous trees in mixed forest landscapes (~ 50% of deciduous forests). Moth abundance was selected in the best models for six among nine bat species. The positive effect of moth abundance on *Barbastella barbastellus* was mediated by vegetation clutter, with dense understory cover likely reducing prey accessibility. Altogether, our findings deepen our understanding of the ecological processes affecting bats in forest landscapes and strengthen the need to consider both landscape context and trophic linkage when assessing the effects of stand-scale compositional and structural attributes on bats.

## Introduction

The amount of forest habitat at larger scales–irrespective of its spatial distribution—is the major factor allowing the maintenance of forest taxa across various biomes and landscapes^1^. The expansion of forest area in many parts of the world, especially in temperate regions^2^, might outline a positive perspective for forest biodiversity conservation. Nevertheless, the carrying capacity of these new forest patches highly depend on their quality^3^ which is partly determined by their structure and composition (here defined as the degree of mixture between coniferous and deciduous tree species). These two forest attributes are known to be key drivers of species diversity and are considered as the most practical biodiversity indicators for forest management planning^4^.

The influence of forest structure on bats has received a lot of attention in the past decades in Europe^5–14^, yet the effects of forest composition have mainly been addressed in relation to bat roost selection^15–17^. While deciduous trees may provide both important resources that enhance bats’ insect prey abundance^18^ and suitable tree-roosts^19^, there is growing evidence that coniferous forests may also represent good foraging and roosting habitats for several bat species^20–24^. As a result, it seems likely that mixing conifers and broadleaves, at both the local and the landscape scales, might enhance bat activity and diversity, through an increase in habitat heterogeneity^25^.

Studies relating bat activity to forest composition have increased recently^22,26,27^ but often lack a multi-scale approach (but see^27^). The landscape matrix around the main forest patch also needs to be of high quality and include several, even small-sized, habitat patches to allow landscape-scale complementation and/or supplementation of resources vital to many animal organisms^28–30^. There is consequently a need for a multiscale approach when assessing animal responses to forest composition and configuration, especially for mobile vertebrates such as bats that use different habitats to fulfil their needs, even on a daily basis^27,31–33^. Bats are especially sensitive to both fine-scale and coarse-grained variation in forest composition^34^ because of the large range of various resources they need to exploit both daily and across their annual life cycles, i.e. for roosting, commuting, foraging, swarming, and mating^35^.

To go beyond the basic comparison between pure deciduous and pure coniferous stands, it is crucial to investigate how bats respond to deciduous-coniferous mixture gradients at multiple spatial scales and how these scales may interact to better understand the ecological processes affecting bats in forests^36^. Another process that may explain the effects of forest attributes on bat activity is the bat-prey relationships^36^, which are expected to be complex and to strongly vary between species^37,38^. Moths constitute the main prey items of many temperate insectivorous bats^39^. Several studies highlighted the importance of deciduous tree cover within coniferous plantations in enhancing moth diversity and abundance^40,41^, which may explain the positive selection of deciduous forests by many forest bat species. Furthermore, stand structure may influence how prey are distributed within forests and most importantly, how easily accessible they are for bats^42^. In fact, vegetation clutter may on the one hand enhance insect abundance and diversity but on the other hand could reduce both prey accessibility and foraging efficiency as well as manoeuvrability for bats^6,43^.

Here, we aimed at testing the assumption that (i) forest composition has interacting effects on bats between stand and landscape scales; and (ii) stand structure mediates prey abundance effects on bat activity. Our first objective was to assess changes of species-specific bat activity to a gradient of deciduous-conifer mixture at both stand and landscape scales. Based on the habitat complementation hypothesis which stipulates that species may require a number of complementary, non-substitutable resources in the landscape that are located in different habitat types^29^, we predicted that bat activity would increase locally (i.e. at the stand level) with deciduous tree cover in conifer-dominated landscapes, and increase with coniferous tree cover in deciduous-dominated landscapes. As bats require multiple resources provided by different forest types (i.e. stands of different species composition and structure) for foraging, we expected higher bat activity in rarer forest habitats at the landscape level that would offer non-substitutable key resources (e.g. different prey types). Our second objective was to determine the additional effect of prey abundance on bats together with forest composition and structure, and to ask if stand structure could mediate the effects of prey abundance on bat activity. We predicted that (i) the effects of prey abundance on bat activity would be more pronounced in stands with low vegetation clutter; and (ii) dietary-specialized species would be more affected by prey abundance than diet generalists.

## Results

### Bat activity and moth abundance

We recorded 134,353 and 55,023 bat sequences (i.e. 5-s recordings) that could be reliably attributed to a species or species group with a maximum error risk tolerance of 50% and 10%, respectively. We detected and confirmed the presence of 12 species or species groups (range: 1–10 per site and per night) across the 42 sampling sites on 168 detector nights. The most active species (or species groups) were *Pipistrellus pipistrellus*, *Pipistrellus pygmaeus*/*Miniopterus schreibersii* and *Nyctalus* spp. (Supplementary Table S1). We captured a total of 16,086 moths that comprise 7374 small individuals (mean per site: 176 individuals; range: 13–643), 4487 medium-sized individuals (mean per site: 107 individuals; range: 24–658), and 4225 large individuals (mean per site: 101 individuals; range: 11–754).

### Effects of forest composition on bats

**Figure 1:**
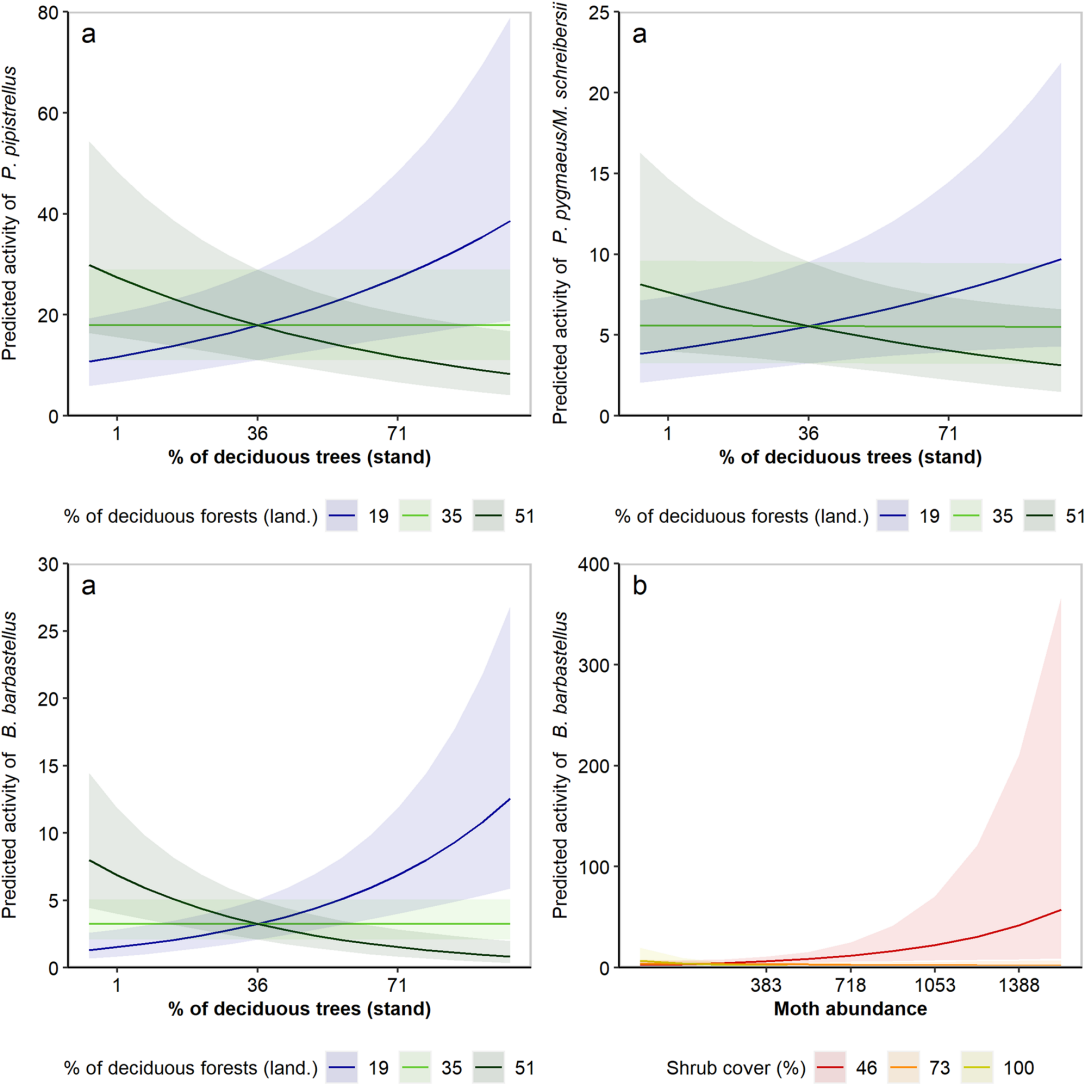
Predicted responses of (**a**) bats to stand composition at each level (mean, mean-SD, mean + SD) of landscape composition; and (**b**) *B. barbastellus* to moth abundance at each level (mean, mean-SD, mean + SD) of shrub cover. Stand composition corresponds to the percentage cover of deciduous trees within stand while landscape composition was inferred using the proportion of deciduous forests within the forested landscape area at 1 km radius scale. Predictions from GLMMs are represented by the solid lines with 95% confidence intervals indicated in the same colour.

For all species except *Myotis nattereri*, forest composition at either stand or landscape scale was retained in the best candidate models after model selection (Supplementary Table S2). Our models indicated that the proportion of coniferous trees at the stand scale positively affects *Plecotus* spp. (Table 1). The activity of *Nyctalus* species group was enhanced in stands located in mixed forest landscapes. We found a similar pattern for *Rhinolophus hipposideros* but this result was not confirmed with acoustic data identified at the 10% error risk tolerance (Supplementary Table S3). The two-way interaction of forest composition at the stand (i.e. proportion of deciduous trees within stand) and landscape scales (i.e. proportion of deciduous forests within the forested landscape area at 1 km radius scale) was selected in the best models for *Barbastella barbastellus*, *Hypsugo savii*, *Nyctalus* spp., *P. pipistrellus*, and *P. pygmaeus*/*M. schreibersii* activity (Table 1). This interaction was, however, non-significant for *H. savii* activity when using the acoustic data identified at the 10% error risk tolerance (see Supplementary Table S3). The spotlight analysis revealed consistent patterns of this interaction effect on *B. barbastellus*, *P. pipistrellus*, and *P. pygmaeus*/*M. schreibersii* activity, even though the signal detected was weaker for *P. pygmaeus*/*M. schreibersii* (Supplementary Table S4). In mixed forest landscapes (i.e. ~ 50% of deciduous forests), bat activity was higher in stands with a higher proportion of coniferous trees. Conversely, in conifer-dominated landscape (> 80% of coniferous forests), these bat species were more active in stands with a higher proportion of deciduous trees (Fig. 1). No significant trend was detected for *Nyctalus* spp. (Supplementary Table S4).

**Table 1:** Standardized, model-averaged parameter estimates with associated standards errors (SE) and 85% confidence intervals of the best GLMMs (Δ*AICc* < 2) relating the effects of forest composition, landscape structure, stand structure, moth abundance on bat activity.

Response variable	Explanatory variable	Estimate (± SE)	Lower 85	Upper 85
*B. barbastellus* ^¥^ (nb)	Stand composition:Landscape composition*	**−** **0.88 (± 0.23)**	**−** **1.21**	**−** **0.55**
	Small-sized moth abundance:Shrub cover*	**−** **0.79 (± 0.21)**	**−** **1.09**	**−** **0.49**
	Julian day*	**0.70 (± 0.21)**	**0.40**	**1.00**
*H. savii* (nb)	**Stand composition:Landscape composition**	**−** **0.34 (± 0.18)**	**−** **0.60**	**−** **0.08**
	Small-sized moth abundance:Canopy openness	**−** 0.19 (± 0.40)	**−** 0.77	0.39
	Temperature*	**0.88 (± 0.24)**	**0.53**	**1.23**
*M. nattereri* (p)	Canopy openness*	**−** **0.36 (± 0.15)**	**−** **0.58**	**−** **0.14**
	Julian day*	**0.51 (± 0.21)**	**0.21**	**0.81**
*Nyctalus* spp. (p)	Stand composition	0.10 (± 0.12)	**−** 0.07	0.27
	Landscape composition*	**0.36 (± 0.18)**	**0.10**	**0.62**
	Stand composition:Landscape composition*	**−** **0.17 (± 0.10)**	**−** **0.31**	**−** **0.03**
	Landscape structure	0.20 (± 0.17)	**−** 0.04	0.44
	Medium-sized moth abundance	0.23 (± 0.16)	0.00	0.46
	Live tree basal area*	**−** **0.21 (± 0.12)**	**−** **0.38**	**−** **0.04**
	Temperature*	**0.36 (± 0.07)**	**0.26**	**0.46**
	Humidity*	**0.18 (± 0.05)**	**0.11**	**0.25**
*P. kuhlii/nathusii* (qp)	**Landscape composition**	**0.44 (± 0.25)**	**0.08**	**0.80**
	Landscape structure	**−** 0.14 (± 0.26)	**−** 0.51	0.23
	Small-sized moth abundance:Canopy openness	0.01 (± 0.31)	**−** 0.44	0.46
	Canopy openness*	**0.67 (± 0.16)**	**0.44**	**0.90**
	Temperature*	**0.45 (± 0.21)**	**0.15**	**0.75**
*P. pipistrellus* ^a^ (qp)	Stand composition:Landscape composition*	**−** **0.37 (± 0.12)**	**−** **0.54**	**−** **0.20**
	Landscape structure*	**−** **0.36 (± 0.24)**	**−** **0.71**	**−** **0.01**
	Live tree basal area*	**−** **0.64 (± 0.22)**	**−** **0.96**	**−** **0.32**
	**Dead tree basal area**	**0.39 (± 0.17)**	**0.15**	**0.63**
	Shrub cover*	**−** **0.95 (± 0.21)**	**−** **1.25**	**−** **0.65**
	Canopy openness*	**−** **0.37 (± 0.21)**	**−** **0.67**	**−** **0.07**
	Humidity*	**−** **0.15 (± 0.04)**	**−** **0.21**	**−** **0.09**
*P. pygmaeus*/*M. schreibersii* ^b^ (nb)	Stand composition:Landscape composition*	**−** **0.34 (± 0.16)**	**−** **0.57**	**−** **0.11**
	Small-sized moth abundance:Shrub cover	**−** 0.22 (± 0.23)	**−** 0.55	0.11
	Shrub cover*	**−** **0.52 (± 0.22)**	**−** **0.84**	**−** **0.20**
	Humidity*	**−** **0.27 (± 0.10)**	**−** **0.41**	**−** **0.13**
*Plecotus* spp. ^¥^ (p)	Stand composition*	**−** **0.74 (± 0.22)**	**−** **1.06**	**−** **0.42**
	Total moth abundance:Shrub cover	0.17 (± 0.37)	**−** 0.36	0.70
	Julian day*	**−** **0.43 (± 0.17)**	**−** **0.67**	**−** **0.19**
*R. hipposideros* (nb)	**Landscape composition**	**0.39 (± 0.25)**	**0.03**	**0.75**
	Canopy openness*	**0.49 (± 0.24)**	**0.14**	**0.84**
	Julian day*	**0.64 (± 0.27)**	**0.25**	**1.03**

Results on bat activity are presented for the 50% maximum error risk tolerance applied to the identification of bat sequences and resulting bat activity (i.e. number of 1-min intervals with ≥ 1 bat sequences). Variables in bold represent influential variables for which 85% CI did not overlap zero. Stand composition corresponds to the percentage cover of deciduous trees within stand while landscape composition was inferred using the proportion of deciduous forests within the forested landscape area at 1 km radius scale. Family distribution of the GLMMs is given in brackets.

^a^Only recordings from the Batlogger were considered, ^b^ One outlier was removed.

Distribution family: p Poisson, qp quasi-Poisson, nb negative binomial.

^¥^Moth-feeding specialist (Supplementary Table S6).

*Influential variable showing consistent pattern between the two separate sets of bat activity (see Supplementary Table S3 regarding results on acoustic data with maximum error risk tolerance of 10%).

### Effects of stand structure on bats

**Figure 2:**
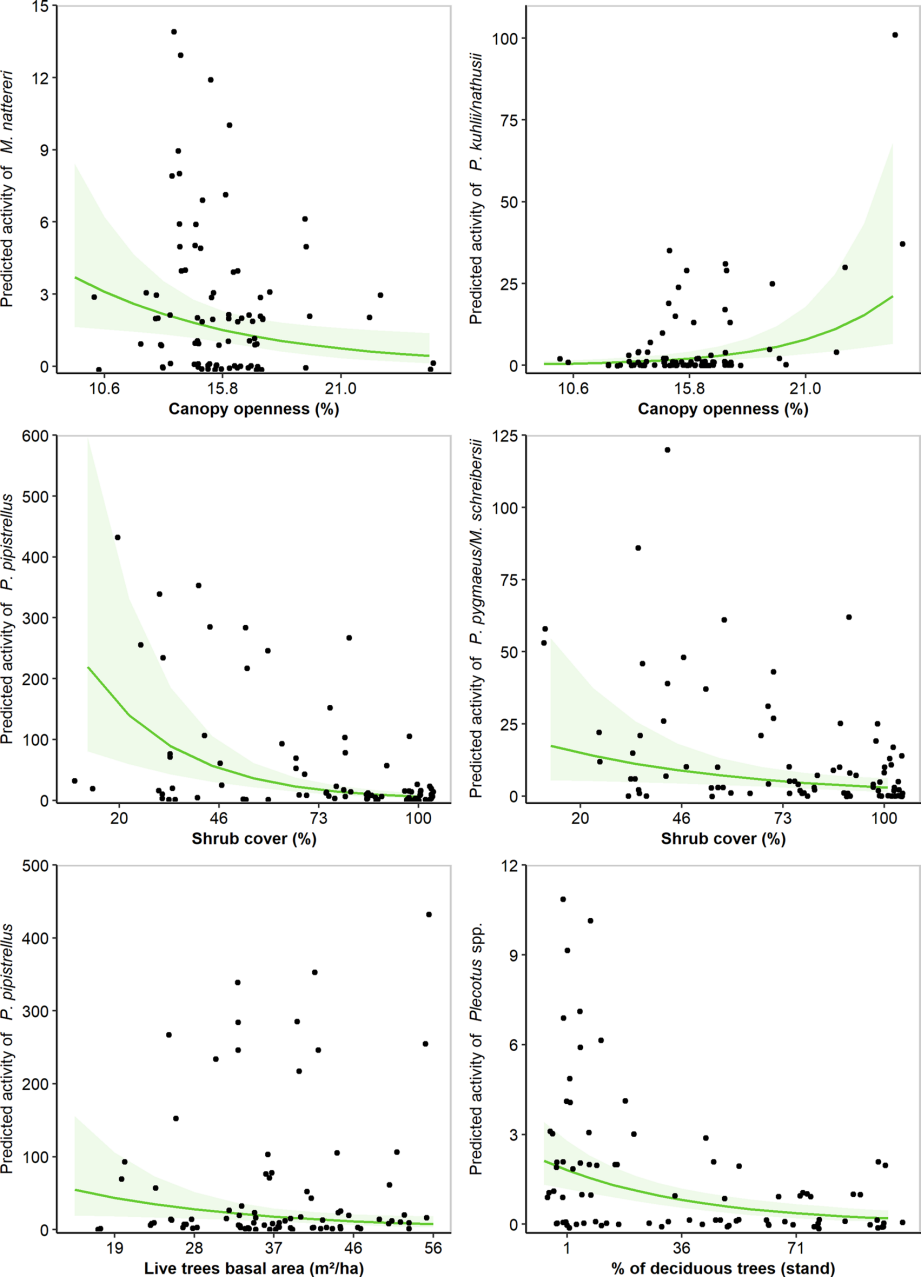
Predicted responses of bats in relation to stand structure and composition. Predictions from GLMMs are represented by the green solid lines with 95% confidence intervals indicated in light green. The raw data are indicated with fill black circles.

Stand structure had contrasting effects on bats (Table 1). For *M. nattereri*, only one best candidate model was retained after model selection and it included the effect of stand structure with 34% of AICc weight. While *M. nattereri* and *P. pipistrellus* activity decreased substantially with canopy openness, we found a reverse pattern for *R. hipposideros* and *Pipistrellus kuhlii/nathusii* (Fig. 2). The activity of *P. pipistrellus* and *P. pygmaeus*/*M. schreibersii* decreased with increasing shrub cover (Fig. 2). Live tree basal area had a negative effect on *P. pipistrellus* and *Nyctalus* spp. whereas *P. pipistrellus* responded positively to basal area of large standing and lying dead trees. Nevertheless, only the effect of live tree basal area on *P. pipistrellus* was confirmed when using acoustic data identified at 10% error risk tolerance (Supplementary Table S3).

### Effects of moth abundance on bats

Candidate models including moth abundance on its own or alongside forest composition and stand structure were considered as the most parsimonious ones for *B. barbastellus*, *H. savii, Nyctalus* spp., *P. kuhlii/nathusii*, *P. pygmaeus/M. schreibersii*, and *Plecotus* spp. (Supplementary Table S2). The best models obtained for *B. barbastellus* revealed a negative interaction between shrub cover and total moth abundance on its activity (Table 1). More specifically, the spotlight analysis indicated that *B. barbastellus* activity was enhanced by moth abundance only in stands with low shrub cover (< 50%; Fig. 2). We also found a negative relationship between *B. barbastellus* activity and moth abundance in stands fully covered by shrubs (Supplementary Table S4). Regarding the other species, the interaction term between moth abundance and stand structure was not significant as the 85% confidence intervals of the model-averaged estimates overlapped zero.

## Discussion

There is growing evidence that mixed forest stands could represent optimal habitats for bats^25^. To go further, our results indicated that the positive effects of mixing conifer and deciduous trees at the stand level on bat activity changed according to the dominant forest composition in the surrounding landscape. We found that *B. barbastellus*, *P. pipistrellus* and *P. pygmaeus*/*M. schreibersii* were more active in deciduous-dominated stands compared to coniferous-dominated ones only in landscapes where coniferous forests predominate (> 80% of coniferous forests). On the other hand, these bat species were more active in coniferous-dominated stands only in mixed forest landscapes (~ 50% of deciduous forests). Thus, as expected and accurately predicted by the habitat complementation hypothesis^29^, bat activity was higher in stands that were rarer at the landscape level and that potentially provided non-substitutional resources to bats for both foraging and roosting. This scale-dependent effect of forest composition on bats is likely a combination of bat foraging preferences and optimization of prey resources provided by mixed and structurally heterogeneous forests^27^. More heterogeneous forest landscapes are actually more beneficial to mobile vertebrates such as bats, boosting several processes including resource complementation, microhabitat supply, and niche diversity^44,45^. For instance, there is good evidence that moth abundance and diversity increase with tree species diversity in forests^46^. Another non-exclusive hypothesis to explain our results is that bat species may concentrate their activity and make a more intensive use of higher-quality habitats (here mature beech stands) present in less favourable landscapes (i.e. concentration effect) while a dilution effect may occur in more favourable landscapes^12^. However, this would assume that mature coniferous forests represent lower-quality habitats for bats in our study area which is not confirmed by local radiotracking studies^47^. As highlighted by other studies^25,26^, mature coniferous forests should receive more attention in Europe as they represent valuable habitats for bats. Finally, it is important to point out that the survey was conducted during the lactation period and that it is very likely that bat responses to forest composition and structure may vary between seasons^22^.

Amongst the local attributes assessed, canopy openness, shrub cover, and live tree basal area were key factors affecting bat activity in forests. Taken together, these variables are the most descriptive of vegetation clutter along the vertical and horizontal profiles of forest stand structure. As expected, *P. kuhlii/nathusii,* and *P. pygmaeus*/*M. schreibersii* were negatively affected by vegetation clutter. These edge-specialist foragers are known to avoid foraging or commuting in highly cluttered situations^6,9,12,37^. Stands with open canopy and low shrub cover may therefore represent optimal foraging habitats for these species. *R. hipposideros* was also found to be more active in stands with more open canopy but this could be due to a better detection probability of this species that emits high frequency echolocation calls (but see ^48^ regarding the weak effect of foliage density on call attenuation). Live tree basal area was negatively associated with *P. pipistrellus* and *Nyctalus* spp. activity, thus confirming that these bats avoid foraging or commuting in dense stands^9,21^. Our results also demonstrated that *P. pipistrellus* activity was negatively affected by canopy openness. However, this result should be interpreted with caution as we were not able to use data recorded at the sub-canopy level for this species. *M. nattereri* responded positively to vegetation clutter since its activity was higher in stands with closed canopy. This species is well adapted for slow manoeuvrable flight close to vegetation (low wing loading and low wing aspect ratio^49^) and is considered as a foliage-gleaning species that brushed the vegetation with its tail membrane to capture its prey^50^. In line with previous research^14,41^, *M. nattereri* seems to benefit from dense vegetation clutter in forests.

There is strong support for bat activity in forest ecosystems being more related to habitat structure than to prey abundance^37,38,51,52^. Yet, the interactive effect between prey abundance and habitat structure has been largely overlooked to date. In our study, models that included prey abundance in combination with stand structure were selected for six among nine species, but their interaction effect was not significant for most taxa, except for *B. barbastellus*. It confirms previous findings that moth biomass is a key driver of foraging site choice by *B. barbastellus*^53^. Our results indicated that the activity of *B. barbastellus* was positively related to moth abundance only in stands where the proportion of shrub cover was low (< 50%). Following Rainho, et al.^42^, we suggest that dense vegetation clutter may decrease access to prey for this moth-feeding bat species that mainly forages at ground level during the lactation period^47,54^ and highly relies on prey-rich habitat patches for foraging^55^. *B. barbastellus* has pointed wings making its flight less manoeuvrable in highly-cluttered habitats compared to other cluttered-adapted species with rounded wings^49^. Interestingly, the influence of vegetation clutter on bat-prey relationships was only significant for the most dietary-specialized bat species feeding on moths. However, as we did not sample the wide range of prey types that bats can feed on, the effects of vegetation clutter on bat-prey relationships for diet-generalist species remain to be explored.

In terms of bat conservation in mountain forests, our results strengthen the potential role of forest managers through the manipulation of stand structure to optimize both bat activity and insect abundance. Given that the responses of bats to three-dimensional structure of forest stands vary between species^6,8,43^, a ‘one-size-fits-all’ approach should be avoided. Rather, forestry practices favouring a mosaic of stand structure with varying degree of vegetation clutter within stands should be encouraged as they are likely to benefit the whole forest bat community^56^. Moreover, we strongly advocate for identifying hotspots of moth abundance to be targeted as main conservation management areas for bats^57,58^ since changes in moth abundance may have a cascading effect on bat activity patterns. Regarding the degree of mixture between coniferous and deciduous tree species, forest management strategies aiming at mixing tree species at the landscape scale^59^ are likely to benefit bats as such strategies may assure that bat prey would be always abundant within the landscape through a spatio-temporal complementation process between habitat types. Our results also highlight the crucial need of adopting a multi-scale approach when designing and implementing forest management strategies that promote biodiversity conservation^60^. For instance, we demonstrated that the beneficial effects of mixing tree species at the stand scale on bats largely depend on the forest composition at the landscape scale. This complements previous recommendations suggesting that increasing structural heterogeneity at the landscape scale would enhance bat activity^21^.

Overall, our findings suggest being cautious when interpreting responses of mobile taxa to forest composition and structure based on measurement at the stand scale alone. We strongly recommend future studies to account for both landscape context and trophic linkage when assessing the effects of stand-scale compositional and structural attributes on bats. Finally, we believe that recommendations to enhance biodiversity in forests should not be solely based on the responses of a single taxon. Indeed, no taxon can be taken to represent a generalised indicator of biodiversity^61^ since cross-taxon congruence is often statistically weak^62^ and rarely consistent^63^. Moreover, taxa may respond differently to the same array of stand features^64^. We therefore urge further research to assess the responses of different taxa to forest composition at multiple spatial scales.

## Material and methods

### Study area

**Figure 3:**
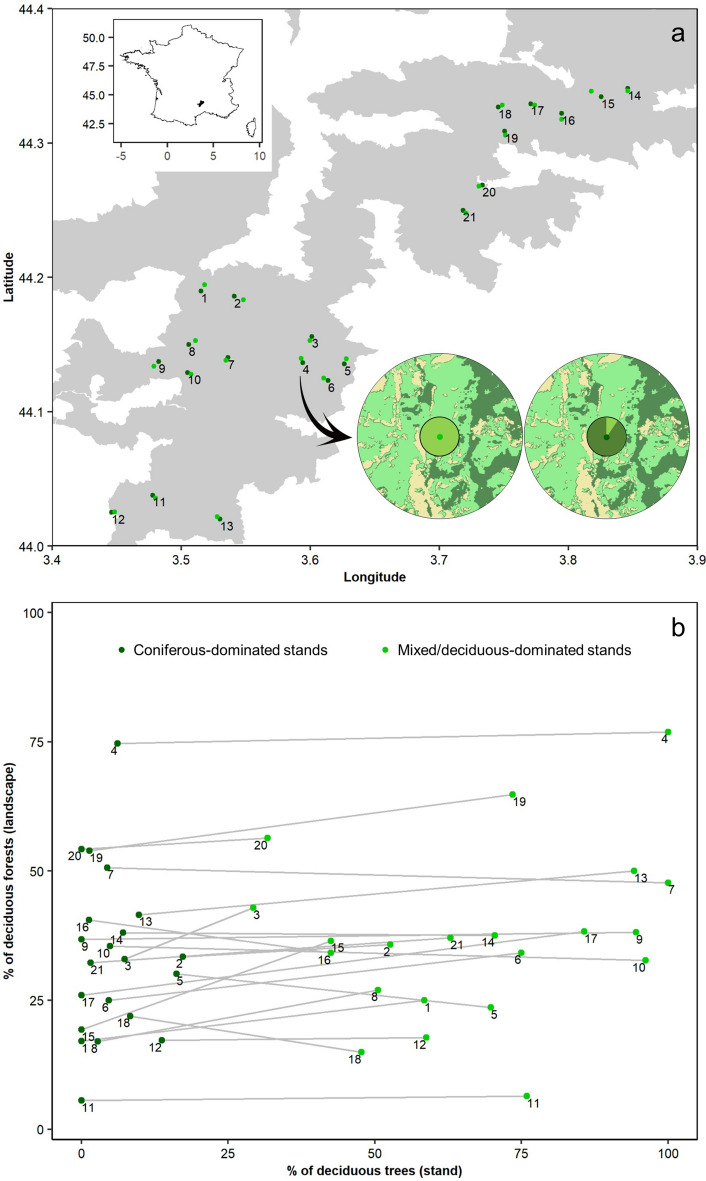
Study design. (**a**) Location of the 21 paired stands in the Cévennes Biosphere Reserve and National Park. Each number represents a pair of one coniferous-dominated stand and one mixed/deciduous-dominated stand. The core zone of the study area is represented in grey. Schematic representation (not in scale) of the land cover within 1-km radius buffer of one pair is depicted (dark green: coniferous forests; olive green: deciduous forests; beige: others) with a pie chart at its centre representing the percentage of deciduous trees (light green) vs. coniferous trees (dark green) at the stand scale. (**b**) Distribution of the sampled stands along a gradient of deciduous-conifer mixture at both stand of and landscape levels (see material and methods for more details). Stands of the same pair have the same number and are linked with a grey line. The map was created using ArcGIS v10.2 (ESRI, Redlands, California, USA; https://desktop.arcgis.com/fr/arcmap/).

The study was conducted in the Cévennes Biosphere Reserve and National Park (c. 44° 19′ N, 3° 35′ E) located in the south of France (Fig. 3). This area has a mountain climate with a strong Mediterranean influence. We focused on three large forest massifs located within the central zone of the National Park, namely the Aigoual forest (16,123 ha, 347–1567 m a.s.l.), the Bougès forest (3187 ha; 550–1421 m a.s.l.), and the Fontmort forest (1768 ha; 658–1003 m a.s.l.). These three public forests are managed by the National Forest Office and have a mixture of pure deciduous, pure coniferous, and mixed tree stands. More than 50 tree species occur within the study area. The most abundant ones occurring in the three forest massifs are European beech (*Fagus sylvatica*; present in 35.9% of the public forested surface area), fir (*Abies* spp*.*; 12.9%, mainly silver fir *Abies alba*), Norway spruce (*Picea abies*; 11.7%), and Scots pine (*Pinus sylvestris*; 7.6%).

### Sampling design and site selection procedure

We implemented a paired-sampling design, each pair consisting of (i) one coniferous-dominated stand and (ii) one deciduous-dominated or mixed stand, located along a landscape composition gradient. Stand selection was conducted at three spatial scales. At the stand scale (i.e. forest management unit), we first selected, from the National Forest Office database, mature stands (presence of standing/lying dead trees and very large trees with diameter at breast height (DBH) > 60 cm) dominated by *Fagus sylvatica*, in association with either *Abies alba* or *Picea abies*. Then, we matched the selected stands with mature coniferous stands (> 80% of coniferous trees) dominated by *Abies alba* or *Picea abies* that were located at the same altitude (mean altitude differences: 33.98 m, range: 0–157 m) and within roughly 1 km range (mean distance: 497 m, range: 262–1067 m). Stands of the same pair occurred in a relatively similar landscape context with respect to forest cover and proportion of deciduous forest within the forested landscape at different spatial scales (Fig. 1; Supplementary Table S5). At the scale of the stand pairs, we aimed at selecting pairs that were separated by a minimum distance of 2 km (mean distance: 2332 m, range: 1900–4377 m) to ensure spatial independence between pairs. Finally, at the landscape scale, we calculated the amount of forest cover and its composition within 1 km radius buffer around the sampling points (i.e. centre of the stands where bat detectors and light-traps were placed) using the CES OSO land cover data 2018 (10 m resolution; osr-cesbio.ups-tlse.fr/ ~ oso/) on ArcGIS Desktop v10 (ESRI, Redlands, CA, USA). We defined the spatial grain of the landscape by considering the mean daily foraging movement of European forest bat species while maximizing the targeted landscape composition gradient^44^. Landscape-scale mixture of deciduous and coniferous tree species (hereafter referred to as “landscape composition”) was inferred using the proportion of deciduous forests within the forested landscape area. In total, we selected 21 pairs that were located in forest-dominated landscapes (> 50% of forest cover within 1 km radius around the sampling points) along a gradient of proportion of deciduous forests ranging from 6 to 77% of total forest cover (median = mean = 35%; Fig. 1). Within stands, sampling points were located at least 50 m away from the stand edge to avoid biases from potential edge effects.

### Assessment of plot- and landscape-scale variables

We conducted field measurements of structure and composition on fixed-angle plots (~ 30–50 m radius buffer area around the sampling points, depending on location of the largest tree) in July 2019. Using a Bitterlich relascope^65^ we recorded basal area (m^2^/ha) of (i) living deciduous and coniferous trees and (ii) large (diameter at 1.3 m from the thickest end > 40 cm) standing and lying dead trees^64^. Stand composition was inferred using the percentage cover of deciduous trees, calculated with basal area measurements^27^. Cover of deciduous trees ranged from 30 to 100% in plots located within deciduous/mixed stands and from 0 to 20% in plots in coniferous-dominated stands (Fig. 1). To estimate fine-scale canopy openness and understorey shrub cover (vegetation at 1–8 m height), we established two perpendicular transects of 20 m centred on the sampling point and collected the data at 5-m intervals (i.e. at 9 survey points). While we acknowledge that this scale does not represent the species-specific bat detection range, we assumed that the data collected were representative of the whole stand. Canopy openness at each survey point was derived from hemispherical pictures taken at 1.80–2.10 m height using a fished-eye lens 235° connected to a tablet. We then used the “Sky” package^66^ implemented in R v3.6.0^67^ to calculate the percentage of canopy openness. Shrub cover was estimated using the stratiscope method^68^ which consists of noting at each survey point the presence/absence of foliage at 1–8 m height within a virtual vertical cylinder of 50 cm diameter. The cover (%) is then calculated based on the number of points in which the shrub layer was contacted divided by the total number of survey points.

To describe the forest at the landscape scale we calculated the amount of forest cover, number of forest patches, forest edge density, and mean forest patch area. We used the “landscapemetrics” package^69^ and the CES OSO land cover data 2018 to compute these variables within 1 km radius buffer around the sampling points. We chose the same spatial grain as for the computation of the landscape composition (see above).

### Bat echolocation call recording and identification

We conducted bat surveys from 24th June to 24th July 2019 using passive acoustic sampling. We simultaneously surveyed ground and canopy levels by allocating two detectors per site: (i) one Batlogger A/A + (Elekon AG, Lucerne, Switzerland) mounted on a pole at 1.70 m height; and (ii) one AudioMoth (https://www.openacousticdevices.info) mounted up in the sub-canopy (mean height ± SD: 11.02 m ± 2.13) using a pulley system previously installed with a slingshot (Big Shot II, Sherrill Tree, Greensboro, NC). This design has been successful in accounting for the vertical stratification in bat assemblage in forests^70^, and especially in optimizing detection of foliage-gleaning bats foraging high in the tree canopy, such as *Myotis bechsteinii*^54^. Time between the two types of detectors were synchronized, and both detectors were set to record sounds in full spectrum at 384 kHz sampling rate. For Batlogger only (given that AudioMoth did not have any trigger system in 2019), recording was triggered automatically when sounds in the frequency range 8–192 kHz with a signal-to-noise-ratio level above 6 dB were detected. Stands from the same pair were sampled simultaneously, during two consecutive nights, from 30 min before sunset to 30 min after sunrise. This sampling effort represents a good trade-off to adequately assess bat habitat use and characterize local bat assemblages while at the same time maximizing the number of sites surveyed during the same period. We sampled between one and four stand pairs simultaneously during each sampling night. Sampling took place only when weather conditions were optimal for bats to forage, i.e. with no rain, wind speed < 30 km/h, and temperature at sunset > 12 °C. We monitored temperature at night (°C) and relative humidity (%) every 15 min using a data logger (HOBO H08-004-02; accuracy: ± 0.7 °C for temperature and ± 5% for humidity; Onset Computer Corp., Pocasset, MA) shielded within a multiplate meteorological shelter placed at 1.5 m height.

Recordings were cut into 5 s audio sequences using Kaleidoscope v5.1.9 (Wildlife Acoustics, Maynard, MA) and were then analysed using the *Tadarida* software^71^. Based on a random forest algorithm, this software assigns with a confidence index value (from 0 to 1) each sequence containing bat echolocation calls to a bat species. Following recommendations from Barré, et al.^72^ to account for potential automated identification errors^73^, we retained two separate sets of bat sequences: (i) sequences with a score ≥ 0.90 (i.e. data with maximum error risk tolerance of 10%); and (ii) sequences with a score ≥ 0.50 (i.e. data with maximum error risk tolerance of 50%). While the former threshold is very conservative by discarding a high number of false negatives, the latter one allows to increase sample size but is less cautious and may include false positives. Using these two thresholds allowed us to check for result consistency during the statistical analysis process and ensure that biases due to identification errors are minimal. Furthermore, for species having ambiguous calls that are somewhat difficult to identify at species level^74^, we grouped them into different groups, namely: *Plecotus* spp. (*P. auritus* and *P. austriacus*), *Myotis* spp. (all *Myotis* species except *Myotis nattereri*), *Nyctalus* spp. (*N. leisleri* and *N. noctula*), *Pipistrellus kuhlii*/*nathusii*, and *Pipistrellus pygmaeus*/*Miniopterus schreibersii*. It is likely that most individuals of the (i) *P. kuhlii*/*nathusii* group comprised *P. kuhlii* as *P. nathusii* only occurs in the study area during the migration period; and (ii) *P. pygmaeus*/*M. schreibersii* group comprised *P. pygmaeus* as *P. pygmaeus* is very abundant in the study area, capture records of *M. schreibersii* are scarce within the study area, and known large maternity colonies of *M. schreibersii* are located further away from the sampling points (> 10 km for the southernmost sites and > 20 km for the northernmost sites). Finally, we manually checked from the subset of sequences having a maximum error risk tolerance of 10% at least one file per taxon per site and per night to confirm species (or species groups) occurrences (see Supplementary Note 1 for details about species misclassification for recordings from AudioMoths). We used Batsound v4.1.4 (Pettersson Electronik AB, Uppsala, Sweden) to extract several call characteristics and proceed to the identification^74,75^.

Acoustic sampling does not allow us to differentiate individual bats flying around the detector, and therefore we used bat activity as a surrogate of bat abundance. Bat activity per night was measured by counting the number of 1-min intervals in which bat sequences of a given species (or species group) were recorded^9,10^, regardless of the detector type. Using 1-min intervals allowed us to pool the acoustic data from ground and sub-canopy levels to get a single activity index per site and per night and avoiding double counting calls that were simultaneously recorded by the two detectors^6^. Nevertheless, this method does not allow to differentiate commuting from foraging behaviour. Further analyses focused on the activity of the main species (or species groups) recorded over sites. Bat species having a diet dominated by Lepidoptera (i.e. > 65% of diet composition) were considered as a moth-feeding specialist (Supplementary Table S6).

### Moth sampling

Moths were captured using a portable light trap (Heath pattern, 6 W actinic bulb) powered with 12 V lithium batteries. Each stand pair was sampled simultaneously for one night, with light traps operated from dusk until dawn. Attraction radius of such a trap in forest habitats is low (< 30 m)^76^ and sampling points within pairs were sufficiently far away to avoid any interferences between traps. Moths were surveyed within a week following the bat sampling and during similar weather conditions^77^. Full-moon periods (± 3 days) were avoided to minimize potential effects of moonlight on trap efficiency. Moths captured were euthanized using ethyl acetate, and then stored in a -18 °C freezer. Since moth size could matter in bat prey selection^78^ (see also Table S6), we assigned each individual moth to one of the three following size classes based on wingspan: small (wingspan < 30 mm), medium (≥ 30 and ≤ 40 mm), and large (> 40 mm). We further calculated total moth abundance and moth abundance for each size classes at each site and used them as predictors in bat activity models (see below).

### Statistical analysis

We assessed bat responses to (i) deciduous-conifer mixture at both stand and landscape scales, (ii) stand- and landscape-scales variables, and (iii) moth abundance using generalized linear mixed-effects models (GLMMs; “glmmTMB” package^79^) with Poisson, quasi-Poisson, or negative binomial distribution family to handle over-dispersion. Activity of bat species (or species group) (i.e. activity of *B. barbastellus*, *M. nattereri*, *H. savii*, *Nyctalus* spp., *P. kuhlii/nathusii*, *P. pipistrellus*, *P. pygmaeus*/*M. schreibersii*, *Plecotus* spp., and *R. hipposideros*) identified with a maximum error risk tolerance of 50% was introduced as a response variable into the models. We considered site identity nested within stand pair as a random effect to account for both pseudo-replication (bats being surveyed during two consecutive nights) and the paired-sampling design. We conducted several steps prior to building candidate models. First, we independently tested the effects of mean temperature at night, mean relative humidity, and Julian day on bat activity and applied an information theoretic approach to assess the importance of these variables as covariate in our models^80^. Second, we used the same approach to select the most relevant (i) stand-scale variables, i.e. between live tree basal area, dead tree basal area, canopy openness, and shrub cover (Supplementary Note 2 and Table S7), and (ii) variable on moth abundance, i.e. between total abundance, abundance of each size class (large-sized moths, medium-sized moths, and small-sized moths) (Supplementary Note 2 and Table S8). Lastly, due to multicollinearity among variables describing the forest at the landscape scale, we conducted a Principal Component Analysis (PCA) on the log-transformed forest landscape variables. We only considered the first PCA axis (PC1, hereafter referred to as “landscape structure”) given that it accounted for 85% of the variance. High values of PC1 represent highly forested landscapes with few, large forest patches and with low forest edge density (Supplementary Table S9).

For each response variable we built 21 candidate models (including a null one) reflecting our assumptions which stipulate that the effects of (i) stand composition on bats changed with forest composition at the landscape scale; and (ii) local prey abundance on bat activity was mediated by stand structure. We developed five models to investigate the interactive and additive effects of stand and landscape composition and three models to test the effects of stand-scale variables, landscape structure, and moth abundance, respectively. Then, we constructed two models to assess the effects of stand-scale attributes (i) in interaction with moth abundance, and (ii) in combination with landscape structure. Finally, 10 models were developed to consider the interactive and additive effects of stand and landscape composition in addition to either the interaction between stand-scale variables and moth abundance or the combination of stand-scale variables and landscape structure. All explanatory variables were standardized prior to their inclusion in the models to enable comparison of effects sizes and were tested for multicollinearity using both the Spearman’s correlation test and the Variance Inflation Factor (VIF). We did not detect any multicollinearity among predictors (|*r*|< 0.7, VIF < 3)^81^ (Supplementary Fig. S1). Model validation was then conducted using the “DHARMa” package^82^ and diagnostic plots (see Supplementary Note 1 for details about outliers).

We applied an information-theoretic approach using the Akaike Information Criterion corrected for small sample size (*AICc*) to select among the 21 candidate models the most parsimonious ones^80^. When equivalent best models were found (Δ*AICc* < 2), we conducted a model-averaged procedure of models (“AICcmodavg” package^83^), thus accounting for model selection uncertainties^84^. The explanatory variable was considered as significant if the 85% confidence intervals of its estimate did not overlap zero^85,86^. In case of significant interaction between stand and landscape composition or between stand structure and moth abundance, we performed a spotlight analysis to explore the nature of the interaction effect using the “emmeans” package^87^. We investigated the effects of stand composition and moth abundance on bat activity at specific values (mean, mean-SD, mean + SD) of landscape composition and stand structure variable, respectively. Model predictions and spotlight analysis were based on best candidate models having the fewest number of predictors (Figs. 2 and 3). Finally, to check for result consistency and ensure that biases due to acoustic identification errors were minimal, we ran again the best models using bat activity calculated using the lowest level of maximum error risk tolerance (i.e. 10%) as response variable with the appropriate family distribution.

## Supplementary Information

Supplementary Information 1.

Supplementary Information 2.

Supplementary Information 3.

## Data Availability

Data analysed during this study are included in this published article (and its Supplementary Information files). Acoustic recordings are archived and available via the French citizen science programme “Vigie-Chiro” (http://vigienature.mnhn.fr/page/participer-vigie-chiro), at the portal http://vigiechiro.herokuapp.com/.
