# Supplementary material for: Bat responses to changes in forest composition and prey abundance depend on landscape matrix and stand structure
Source: Sci Rep. 2021 May 19;11:10586. doi: 10.1038/s41598-021-89660-z (PMC8134465; doi:10.1038/s41598-021-89660-z)
Supplement: Supplementary file 3 — Supplementary Information 3. [file 41598_2021_89660_MOESM3_ESM.docx]

Bat responses to changes in forest composition and prey abundance depend on landscape matrix and stand structure

**-**

**Supplementary Information**

Jérémy S. P. Froidevaux^1,2,¥,*^, Luc Barbaro^1,3^, Olivier Vinet^4^, Laurent Larrieu^1,5^, Yves Bas^3,6^, Jérôme Molina^1^, François Calatayud^1^, Antoine Brin^7^

^1^ Université de Toulouse, INRAE, UMR DYNAFOR, Castanet-Tolosan, France.

^2^ University of Bristol, School of Biological Sciences, Life Sciences Building, Bristol, UK.

^3^ CESCO, Museum national d'Histoire naturelle, CNRS, Sorbonne-Univ., Paris, France.

^4^ Office National des Forêts (ONF), Agence Etudes Midi-Méditerranée, Montpellier, France.

^5^ CRPF-Occitanie, antenne de Tarbes, Tarbes, France.

^6^ Centre d’Ecologie Fonctionnelle et Evolutive (CEFE), Université de Montpellier, CNRS, EPHE, IRD, Université Paul Valéry, Montpellier, France.

^7^ Université de Toulouse, Ecole d’Ingénieurs de PURPAN, UMR INRAE-INPT DYNAFOR, Toulouse, France.

*Corresponding author: Jérémy S. P. Froidevaux, E-mail: jeremy.froidevaux@stir.ac.uk

^¥^ Present address: Biological and Environmental Sciences, Faculty of Natural Sciences, University of Stirling,

Stirling FK9 4LA, UK.

**Supplementary Notes**

**Supplemental Note 1.** Details on species misclassification and outliers.

**Supplemental Note 2.** Details on model selection.

**Supplementary Tables**

**Supplementary Table S1** Guild- and species-specific bat activity recorded across 42 stands at ground and sub-canopy levels during two consecutive nights.

**Supplementary Table S2** Description of the 20 candidate GLMMs associated to 8 model types relating the effects of forest composition, landscape structure, stand structure, and moth abundance on bat activity.

**Supplementary Table S3** Standardized, model-averaged parameter estimates with associated standards errors (SE) and 85% confidence intervals of the best GLMMs (ΔAICc <2) relating the effects of forest composition, landscape structure, stand structure, moth abundance on bat activity.

**Supplementary Table S4** Results of the spotlight analysis conducted to explore (i) the interactive effects of forest composition at both stand and landscape scales on bat activity; and (ii) the interactive effect between shrub cover and moth abundance on *Barbastella barbastellus* activity.

**Supplementary Table S5** Summary table of the amount of forest cover and proportion of deciduous forest within the forested landscape around the mixed/deciduous-dominated stands and coniferous-dominated stands and across spatial scales.

**Supplementary Table S6** Information on the diet and morphology of the main bat species recorded over sites.

**Supplementary Table S7** Description of the GLMMs relating the effects of stand structure on bat activity.

**Supplementary Table S8** Description of the GLMMs relating the effects of moth abundance on bat activity.

**Supplementary Table S9.** Results from the Principal component analyses (PCA) of variables describing the forest at the landscape scale.

**Supplementary Figure**

**Supplementary Figure S1** Correlation matrix between the explanatory variables.

**Supplementary Note 1**

**Details on species misclassification, outliers, and model convergence issues**

*Misclassification* – We noted that the AudioMoth devices produced some high-frequency noises that were wrongly attributed to *Pipistrellus pipistrellus*, especially when using data with maximum error risk tolerance of 50% (unpublished data). This misclassification was largely attenuated using data with maximum error risk tolerance of 10%. We therefore decided to use recordings from Batloggers only when calculating *P. pipistrellus* activity.

*Outliers* – When screening diagnostic plots for model validation, we detected outliers in *P. pygmaeus*/*M. schreibersii* activity that is likely to indicate a local roost rather than foraging or commuting behaviour, as disproportionately more social calls of *P. pygmaeus* were recorded after sunset at that site during the two survey nights (unpublished data). Thus, this site was disregarded when calculated the activity *P. pygmaeus*/*M. schreibersii* activity.

**Supplementary Note 2**

**Details on model selection**

*Stand structure* – Prior building the 20 candidate models, we assessed independently the relationships between the response variables (i.e. activity of *B. barbastellus*, *M. nattereri*, *H. savii*, *Nyctalus* spp., *P. kuhlii/nathusii*, *P. pipistrellus*, *P. pygmaeus*/*M. schreibersii*, *Plecotus* spp., and *R. hipposideros*) and each stand-scale structural variables (i.e. live tree basal area, dead tree basal area, canopy openness, and shrub cover). We also developed a full model and a null one for comparison. We used the same model structure as described in *Material and methods*. We applied an information-theoretic approach using the Akaike Information Criterion corrected for small sample size (AICc) to select the most parsimonious ones. When several models were identified as equivalent (ΔAICc<2), we chose the one having the fewest number of parameters and the highest AICc weight. The explanatory variables retained were then included in the set of candidate models related to stand structure (Supplementary Table S7).

*Moth abundance* – For selecting the most relevant variable relating to moth abundance, we conducted two different approaches depending on the species or species group considered. For taxa that are known to mainly feed on small-size prey insects i.e. *H. savii*, *P. kuhlii/nathusii*, *P. pipistrellus*, *P. pygmaeus*/*M. schreibersii* and *R. hipposideros* (see Supplementary Table S6), we selected the abundance of small-sized moths to be included in our candidate models related to prey abundance. For the other taxa (i.e. *B. barbastellus*, *M. nattereri*, *Nyctalus* spp. and *Plecotus* spp.), we included sequentially total moth abundance and abundance for each of the three size classes (small, medium, and large) in four different models for each species and compared model fit using change in *AICc*. When several models were identified as equivalent (ΔAICc<2), we chose the one having the fewest number of parameters and the highest AICc weight. The explanatory variables retained were then included in the set of candidate models related to prey abundance (Supplementary Table S8).

**Table S1.** Species-specific bat activity recorded across 42 stands at ground and sub-canopy levels during two consecutive nights. Mean bat activity (i.e. mean number of 1-min intervals where bat sequences were recorded during a night) ± SD is given for two levels of maximum error risk tolerance (10% and 50%) applied to the identification of bat sequences and resulting bat activity (see material and methods for more details).

| **Response variable** | **10% error risk tolerance** | | **50% error risk tolerance** | |
| --- | --- | --- | --- | --- |
|  | Act. Night 1 | Act. Night 2 | Act. Night 1 | Act. Night 2 |
| *Barbastella barbastellus* | 6.79 (±10.15) | 7.43 (±14.51) | 8.00 (±12.06) | 8.67 (±16.88) |
| *Hypsugo savii* | 1.48 (±4.18) | 4.79 (±18.69) | 2.76 (±5.63) | 7.86 (±26.04) |
| *Myotis nattereri* | 1.71 (±2.31) | 2.38 (±2.76) | 2.10 (±2.74) | 2.95 (±3.43) |
| *Nyctalus* spp. | 12.45 (±12.88) | 12.90 (±14.86) | 16.21 (±14.68) | 17.79 (±18.46) |
| *Pipistrellus kuhlii*/*nathusii* | 0.79 (±3.68) | 1.88 (±9.46) | 5.14 (±10.49) | 6.71 (±16.79) |
| *Pipistrellus pipistrellus*^a^ | 49.48 (±98.60) | 56.60 (±94.33) | 52.50 (±99.51) | 60.21 (±96.14) |
| *Pipistrellus pygmaeus/Miniopterus schreibersii*^b^ | 5.83 (±11.63) | 8.88 (±20.02) | 10.76 (±14.49) | 16.83 (±26.26) |
| *Plecotus* spp. | 0.26 (±0.83) | 0.36(±1.01) | 1.60 (±2.33) | 1.48 (±2.40) |
| *Rhinolophus hipposideros* | 1.48 (±2.47) | 1.24 (±1.76) | 3.40 (±6.95) | 4.79 (±17.51) |

^a^ Only recordings from the Batlogger were considered, ^b^ One outlier was removed.

**Table S2.** Description of the 20 candidate GLMMs associated to 8 model types relating the effects of forest composition, landscape structure, stand structure, and moth abundance on bat activity. The null model is also displayed for comparison. Results on bat activity are presented for the 50% maximum error risk tolerance applied to the identification of bat sequences and resulting bat activity (i.e. number of 1-min intervals with ≥1 bat sequences). Models are ranked in ascending order of AICc and the number of parameters (K), AICc weight (ωi), cumulative weight (cum. ωi) are given for each model. The most parsimonious models (ΔAICc < 2) are displayed in bold. Stand composition corresponds to the percentage cover of deciduous trees within stand while landscape composition was inferred using the proportion of deciduous forests within the forested landscape area at 1 km radius scale.

1. *B. barbastellus*

| **Model type** | **Model** | **K** | **AICc** | **ΔAICc** | **ω_i_** | **cum. ωi** |
| --- | --- | --- | --- | --- | --- | --- |
| **Forest composition+Stand+Prey** | **Stand composition:Landscape composition + Stand composition + Landscape composition + Moth abundance:Shrub cover + Moth abundance + Shrub cover + Julian day** | **11** | **452.84** | **0.00** | **0.64** | **0.64** |
| **Forest composition+Stand+Prey** | **Stand composition:Landscape composition + Moth abundance:Shrub cover + Moth abundance + Shrub cover + Julian day** | **9** | **454.01** | **1.18** | **0.35** | **0.99** |
| Forest composition+Stand+Landscape | Stand composition:Landscape composition + Shrub cover + Landscape structure + Julian day | 8 | 462.94 | 10.10 | 0.00 | 0.99 |
| Forest composition+Stand+Landscape | Stand composition:Landscape composition + Stand composition + Landscape composition + Shrub cover + Landscape structure + Julian day | 10 | 462.97 | 10.13 | 0.00 | 1.00 |
| Stand | Shrub cover + Julian day | 6 | 466.00 | 13.16 | 0.00 | 1.00 |
| Forest composition+Stand+Prey | Landscape composition + Moth abundance:Shrub cover + Moth abundance + Shrub cover + Julian day | 9 | 466.23 | 13.39 | 0.00 | 1.00 |
| Stand+Prey | Moth abundance:Shrub cover + Moth abundance + Shrub cover + Julian day | 8 | 466.36 | 13.52 | 0.00 | 1.00 |
| Stand+Landscape | Landscape structure + Shrub cover + Julian day | 7 | 467.64 | 14.80 | 0.00 | 1.00 |
| Forest composition+Stand+Landscape | Landscape composition + Shrub cover + Landscape structure + Julian day | 8 | 467.70 | 14.86 | 0.00 | 1.00 |
| Forest composition+Stand+Prey | Stand composition + Landscape composition + Moth abundance:Shrub cover + Moth abundance + Shrub cover + Julian day | 10 | 468.80 | 15.96 | 0.00 | 1.00 |
| Forest composition+Stand+Prey | Stand composition + Moth abundance:Shrub cover + Moth abundance + Shrub cover + Julian day | 9 | 468.85 | 16.01 | 0.00 | 1.00 |
| Forest composition+Stand+Landscape | Stand composition + Shrub cover + Landscape structure + Julian day | 8 | 470.09 | 17.25 | 0.00 | 1.00 |
| Forest composition+Stand+Landscape | Stand composition + Landscape composition + Shrub cover + Landscape structure + Julian day | 9 | 470.19 | 17.35 | 0.00 | 1.00 |
| Forest composition | Stand composition:Landscape composition + Julian day | 6 | 470.58 | 17.75 | 0.00 | 1.00 |
| Forest composition | Stand composition:Landscape composition + Stand composition + Landscape composition + Julian day | 8 | 470.75 | 17.91 | 0.00 | 1.00 |
| Forest composition | Stand composition + Julian day | 6 | 472.03 | 19.19 | 0.00 | 1.00 |
| Forest composition | Stand composition + Landscape composition + Julian day | 7 | 472.95 | 20.11 | 0.00 | 1.00 |
| Forest composition | Landscape composition + Julian day | 6 | 473.61 | 20.77 | 0.00 | 1.00 |
| Landscape | Landscape structure + Julian day | 6 | 474.97 | 22.13 | 0.00 | 1.00 |
| Prey | Moth abundance + Julian day | 6 | 475.31 | 22.47 | 0.00 | 1.00 |
| - | Null model | 4 | 477.60 | 24.76 | 0.00 | 1.00 |

1. *H. savii*

| **Model type** | **Model** | **K** | **AICc** | **ΔAICc** | **ω_i_** | **cum. ωi** |
| --- | --- | --- | --- | --- | --- | --- |
| **Forest composition+Stand+Prey** | **Stand composition:Landscape composition + Moth abundance:Canopy Openness + Moth abundance + Canopy Openness + Temperature** | **9** | **343.83** | **0.00** | **0.41** | **0.41** |
| **Stand+Prey** | **Moth abundance:Canopy Openness + Moth abundance + Canopy Openness + Temperature** | **8** | **345.19** | **1.36** | **0.21** | **0.61** |
| Forest composition+Stand+Prey | Stand composition:Landscape composition + Stand composition + Landscape composition + Moth abundance:Canopy Openness + Moth abundance + Canopy Openness + Temperature | 11 | 346.15 | 2.32 | 0.13 | 0.74 |
| Forest composition+Stand+Prey | Stand composition + Moth abundance:Canopy Openness + Moth abundance + Canopy Openness + Temperature | 9 | 346.42 | 2.59 | 0.11 | 0.85 |
| Forest composition+Stand+Prey | Landscape composition + Moth abundance:Canopy Openness + Moth abundance + Canopy Openness + Temperature | 9 | 346.64 | 2.81 | 0.10 | 0.95 |
| Forest composition+Stand+Prey | Stand composition + Landscape composition + Moth abundance:Canopy Openness + Moth abundance + Canopy Openness + Temperature | 10 | 348.28 | 4.45 | 0.04 | 1.00 |
| Forest composition+Stand+Landscape | Stand composition + Canopy Openness + Landscape structure + Temperature | 8 | 354.64 | 10.81 | 0.00 | 1.00 |
| Forest composition+Stand+Landscape | Stand composition + Landscape composition + Canopy Openness + Landscape structure + Temperature | 9 | 356.91 | 13.08 | 0.00 | 1.00 |
| Forest composition+Stand+Landscape | Stand composition:Landscape composition + Stand composition + Landscape composition + Canopy Openness + Landscape structure + Temperature | 10 | 357.89 | 14.06 | 0.00 | 1.00 |
| Forest composition | Stand composition + Temperature | 6 | 357.94 | 14.10 | 0.00 | 1.00 |
| Stand | Canopy Openness + Temperature | 6 | 358.29 | 14.46 | 0.00 | 1.00 |
| Stand+Landscape | Landscape structure + Canopy Openness + Temperature | 7 | 358.68 | 14.85 | 0.00 | 1.00 |
| Forest composition | Stand composition:Landscape composition + Stand composition + Landscape composition + Temperature | 8 | 359.19 | 15.36 | 0.00 | 1.00 |
| Forest composition+Stand+Landscape | Stand composition:Landscape composition + Canopy Openness + Landscape structure + Temperature | 8 | 359.33 | 15.50 | 0.00 | 1.00 |
| Forest composition | Stand composition + Landscape composition + Temperature | 7 | 360.26 | 16.43 | 0.00 | 1.00 |
| Forest composition+Stand+Landscape | Landscape composition + Canopy Openness + Landscape structure + Temperature | 8 | 361.09 | 17.26 | 0.00 | 1.00 |
| Prey | Moth abundance + Temperature | 6 | 362.89 | 19.06 | 0.00 | 1.00 |
| Forest composition | Stand composition:Landscape composition + Temperature | 6 | 365.71 | 21.88 | 0.00 | 1.00 |
| Landscape | Landscape structure + Temperature | 6 | 370.83 | 27.00 | 0.00 | 1.00 |
| Forest composition | Landscape composition + Temperature | 6 | 371.08 | 27.25 | 0.00 | 1.00 |
| - | Null model | 4 | 372.31 | 28.48 | 0.00 | 1.00 |

1. *M. nattereri*

| **Model type** | **Model** | **K** | **AICc** | **ΔAICc** | **ω_i_** | **cum. ωi** |
| --- | --- | --- | --- | --- | --- | --- |
| **Stand** | **Canopy Openness + Julian day** | **5** | **327.13** | **0.00** | **0.34** | **0.34** |
| Stand+Landscape | Landscape structure + Canopy Openness + Julian day | 6 | 329.38 | 2.25 | 0.11 | 0.45 |
| Stand+Prey | Moth abundance:Canopy Openness + Moth abundance + Canopy Openness + Julian day | 7 | 330.01 | 2.88 | 0.08 | 0.53 |
| Prey | Moth abundance + Julian day | 5 | 330.59 | 3.46 | 0.06 | 0.59 |
| Forest composition | Stand composition + Julian day | 5 | 330.75 | 3.62 | 0.06 | 0.64 |
| Forest composition+Stand+Landscape | Landscape composition + Canopy Openness + Landscape structure + Julian day | 7 | 331.45 | 4.32 | 0.04 | 0.68 |
| Forest composition+Stand+Landscape | Stand composition:Landscape composition + Canopy Openness + Landscape structure + Julian day | 7 | 331.71 | 4.58 | 0.03 | 0.72 |
| Forest composition+Stand+Landscape | Stand composition + Canopy Openness + Landscape structure + Julian day | 7 | 331.76 | 4.63 | 0.03 | 0.75 |
| Forest composition | Landscape composition + Julian day | 5 | 331.90 | 4.77 | 0.03 | 0.78 |
| Forest composition | Stand composition:Landscape composition + Julian day | 5 | 331.91 | 4.78 | 0.03 | 0.81 |
| Landscape | Landscape structure + Julian day | 5 | 331.92 | 4.79 | 0.03 | 0.84 |
| Forest composition+Stand+Prey | Landscape composition + Moth abundance:Canopy Openness + Moth abundance + Canopy Openness + Julian day | 8 | 332.14 | 5.01 | 0.03 | 0.87 |
| Forest composition+Stand+Prey | Stand composition + Moth abundance:Canopy Openness + Moth abundance + Canopy Openness + Julian day | 8 | 332.20 | 5.08 | 0.03 | 0.90 |
| Forest composition+Stand+Prey | Stand composition:Landscape composition + Moth abundance:Canopy Openness + Moth abundance + Canopy Openness + Julian day | 8 | 332.27 | 5.14 | 0.03 | 0.92 |
| - | Null model | 3 | 332.39 | 5.26 | 0.02 | 0.95 |
| Forest composition | Stand composition + Landscape composition + Julian day | 6 | 332.95 | 5.82 | 0.02 | 0.97 |
| Forest composition+Stand+Landscape | Stand composition + Landscape composition + Canopy Openness + Landscape structure + Julian day | 8 | 333.88 | 6.75 | 0.01 | 0.98 |
| Forest composition+Stand+Prey | Stand composition + Landscape composition + Moth abundance:Canopy Openness + Moth abundance + Canopy Openness + Julian day | 9 | 334.15 | 7.02 | 0.01 | 0.99 |
| Forest composition | Stand composition:Landscape composition + Stand composition + Landscape composition + Julian day | 7 | 335.33 | 8.20 | 0.01 | 0.99 |
| Forest composition+Stand+Landscape | Stand composition:Landscape composition + Stand composition + Landscape composition + Canopy Openness + Landscape structure + Julian day | 9 | 336.36 | 9.23 | 0.00 | 1.00 |
| Forest composition+Stand+Prey | Stand composition:Landscape composition + Stand composition + Landscape composition + Moth abundance:Canopy Openness + Moth abundance + Canopy Openness + Julian day | 10 | 336.47 | 9.34 | 0.00 | 1.00 |

1. *Nyctalus spp.*

| **Model type** | **Model** | **K** | **AICc** | **ΔAICc** | **ω_i_** | **cum. ωi** |
| --- | --- | --- | --- | --- | --- | --- |
| **Forest composition** | **Landscape composition + Humidity + Temperature** | **6** | **620.35** | **0.00** | **0.15** | **0.15** |
| **Stand** | **Live tree BA + Humidity + Temperature** | **6** | **621.16** | **0.81** | **0.10** | **0.26** |
| **Forest composition+Stand+Landscape** | **Landscape composition + Live tree BA + Landscape structure + Humidity + Temperature** | **8** | **621.36** | **1.02** | **0.09** | **0.35** |
| **Forest composition** | **Stand composition:Landscape composition + Stand composition + Landscape composition + Humidity + Temperature** | **8** | **621.77** | **1.42** | **0.08** | **0.42** |
| **Prey** | **Moth abundance + Humidity + Temperature** | **6** | **622.01** | **1.66** | **0.07** | **0.49** |
| **Forest composition** | **Stand composition + Landscape composition + Humidity + Temperature** | **7** | **622.04** | **1.69** | **0.07** | **0.56** |
| Stand+Prey | Moth abundance:Live tree BA + Moth abundance + Live tree BA + Humidity + Temperature | 8 | 622.67 | 2.32 | 0.05 | 0.61 |
| Forest composition | Stand composition + Humidity + Temperature | 6 | 622.71 | 2.36 | 0.05 | 0.65 |
| Forest composition | Stand composition:Landscape composition + Humidity + Temperature | 6 | 622.77 | 2.42 | 0.05 | 0.70 |
| Forest composition+Stand+Prey | Landscape composition + Moth abundance:Live tree BA + Moth abundance + Live tree BA + Humidity + Temperature | 9 | 622.79 | 2.44 | 0.05 | 0.75 |
| Stand+Landscape | Landscape structure + Live tree BA + Humidity + Temperature | 7 | 622.98 | 2.63 | 0.04 | 0.79 |
| Forest composition+Stand+Prey | Stand composition:Landscape composition + Moth abundance:Live tree BA + Moth abundance + Live tree BA + Humidity + Temperature | 9 | 623.08 | 2.73 | 0.04 | 0.83 |
| Forest composition+Stand+Landscape | Stand composition:Landscape composition + Live tree BA + Landscape structure + Humidity + Temperature | 8 | 623.62 | 3.27 | 0.03 | 0.86 |
| Landscape | Landscape structure + Humidity + Temperature | 6 | 623.62 | 3.28 | 0.03 | 0.89 |
| Forest composition+Stand+Landscape | Stand composition:Landscape composition + Stand composition + Landscape composition + Live tree BA + Landscape structure + Humidity + Temperature | 10 | 623.66 | 3.32 | 0.03 | 0.92 |
| Forest composition+Stand+Landscape | Stand composition + Landscape composition + Live tree BA + Landscape structure + Humidity + Temperature | 9 | 623.87 | 3.52 | 0.03 | 0.94 |
| Forest composition+Stand+Prey | Stand composition + Moth abundance:Live tree BA + Moth abundance + Live tree BA + Humidity + Temperature | 9 | 624.91 | 4.56 | 0.02 | 0.96 |
| Forest composition+Stand+Prey | Stand composition:Landscape composition + Stand composition + Landscape composition + Moth abundance:Live tree BA + Moth abundance + Live tree BA + Humidity + Temperature | 11 | 624.91 | 4.56 | 0.02 | 0.97 |
| Forest composition+Stand+Landscape | Stand composition + Live tree BA + Landscape structure + Humidity + Temperature | 8 | 625.16 | 4.81 | 0.01 | 0.99 |
| Forest composition+Stand+Prey | Stand composition + Landscape composition + Moth abundance:Live tree BA + Moth abundance + Live tree BA + Humidity + Temperature | 10 | 625.29 | 4.95 | 0.01 | 1.00 |
| - | Null model | 3 | 650.21 | 29.86 | 0.00 | 1.00 |

1. *P. kuhlii/nathusii*

| **Model type** | **Model** | **K** | **AICc** | **ΔAICc** | **ω_i_** | **cum. ωi** |
| --- | --- | --- | --- | --- | --- | --- |
| **Stand** | **Canopy Openness + Temperature** | **6** | **382.79** | **0.00** | **0.27** | **0.27** |
| **Forest composition+Stand+Prey** | **Landscape composition + Moth abundance:Canopy Openness + Moth abundance + Canopy Openness + Temperature** | **9** | **384.57** | **1.79** | **0.11** | **0.38** |
| **Forest composition+Stand+Landscape** | **Landscape composition + Canopy Openness + Landscape structure + Temperature** | **8** | **384.62** | **1.83** | **0.11** | **0.48** |
| **Stand+Landscape** | **Landscape structure + Canopy Openness + Temperature** | **7** | **384.62** | **1.83** | **0.11** | **0.59** |
| Stand+Prey | Moth abundance:Canopy Openness + Moth abundance + Canopy Openness + Temperature | 8 | 385.21 | 2.42 | 0.08 | 0.67 |
| Forest composition+Stand+Landscape | Stand composition + Canopy Openness + Landscape structure + Temperature | 8 | 385.52 | 2.73 | 0.07 | 0.74 |
| Forest composition+Stand+Landscape | Stand composition + Landscape composition + Canopy Openness + Landscape structure + Temperature | 9 | 385.99 | 3.20 | 0.05 | 0.79 |
| Forest composition+Stand+Landscape | Stand composition:Landscape composition + Canopy Openness + Landscape structure + Temperature | 8 | 386.27 | 3.49 | 0.05 | 0.84 |
| Forest composition+Stand+Landscape | Stand composition:Landscape composition + Stand composition + Landscape composition + Canopy Openness + Landscape structure + Temperature | 10 | 386.58 | 3.79 | 0.04 | 0.88 |
| Forest composition+Stand+Prey | Stand composition + Moth abundance:Canopy Openness + Moth abundance + Canopy Openness + Temperature | 9 | 386.83 | 4.04 | 0.04 | 0.91 |
| Forest composition+Stand+Prey | Stand composition:Landscape composition + Moth abundance:Canopy Openness + Moth abundance + Canopy Openness + Temperature | 9 | 386.91 | 4.12 | 0.03 | 0.95 |
| Forest composition+Stand+Prey | Stand composition + Landscape composition + Moth abundance:Canopy Openness + Moth abundance + Canopy Openness + Temperature | 10 | 386.96 | 4.18 | 0.03 | 0.98 |
| Forest composition+Stand+Prey | Stand composition:Landscape composition + Stand composition + Landscape composition + Moth abundance:Canopy Openness + Moth abundance + Canopy Openness + Temperature | 11 | 388.28 | 5.49 | 0.02 | 1.00 |
| Forest composition | Stand composition:Landscape composition + Temperature | 6 | 394.40 | 11.61 | 0.00 | 1.00 |
| Forest composition | Landscape composition + Temperature | 6 | 395.40 | 12.61 | 0.00 | 1.00 |
| Landscape | Landscape structure + Temperature | 6 | 395.84 | 13.06 | 0.00 | 1.00 |
| - | Null model | 4 | 396.02 | 13.23 | 0.00 | 1.00 |
| Forest composition | Stand composition + Temperature | 6 | 396.55 | 13.76 | 0.00 | 1.00 |
| Forest composition | Stand composition + Landscape composition + Temperature | 7 | 396.62 | 13.83 | 0.00 | 1.00 |
| Forest composition | Stand composition:Landscape composition + Stand composition + Landscape composition + Temperature | 8 | 396.72 | 13.94 | 0.00 | 1.00 |
| Prey | Moth abundance + Temperature | 6 | 397.06 | 14.27 | 0.00 | 1.00 |

1. *P. pipistrellus*

| **Model type** | **Model** | **K** | **AICc** | **ΔAICc** | **ω_i_** | **cum. ωi** |
| --- | --- | --- | --- | --- | --- | --- |
| **Forest composition+Stand+Landscape** | **Stand composition:Landscape composition + Stand composition + Landscape composition + Dead tree BA + Live tree BA + Shrub cover + Canopy openness + Landscape structure + Humidity** | **13** | **703.08** | **0.00** | **0.50** | **0.50** |
| **Forest composition+Stand+Landscape** | **Stand composition:Landscape composition + Dead tree BA + Live tree BA + Shrub cover + Canopy openness + Landscape structure + Humidity** | **11** | **703.78** | **0.70** | **0.35** | **0.86** |
| Forest composition+Stand+Prey | Stand composition:Landscape composition + Moth abundance:Dead tree BA + Moth abundance:Live tree BA + Moth abundance:Shrub cover + Moth abundance:Canopy Openness + Moth abundance + Dead tree BA + Live tree BA + Shrub cover + Canopy openness + Humidity | 15 | 707.60 | 4.52 | 0.05 | 0.91 |
| Forest composition+Stand+Landscape | Stand composition + Dead tree BA + Live tree BA + Shrub cover + Canopy openness + Landscape structure + Humidity | 11 | 708.82 | 5.75 | 0.03 | 0.94 |
| Forest composition+Stand+Prey | Stand composition:Landscape composition + Stand composition + Landscape composition + Moth abundance:Dead tree BA + Moth abundance:Live tree BA + Moth abundance:Shrub cover + Moth abundance:Canopy Openness + Moth abundance + Dead tree BA + Live tree BA + Shrub cover + Canopy openness + Humidity | 17 | 709.49 | 6.41 | 0.02 | 0.96 |
| Stand+Landscape | Landscape structure + Dead tree BA + Live tree BA + Shrub cover + Canopy openness + Humidity | 10 | 710.27 | 7.19 | 0.01 | 0.97 |
| Stand | Dead tree BA + Live tree BA + Shrub cover + Canopy openness + Humidity | 9 | 710.43 | 7.35 | 0.01 | 0.99 |
| Forest composition+Stand+Landscape | Stand composition + Landscape composition + Dead tree BA + Live tree BA + Shrub cover + Canopy openness + Landscape structure + Humidity | 12 | 711.53 | 8.45 | 0.01 | 0.99 |
| Forest composition+Stand+Landscape | Landscape composition + Dead tree BA + Live tree BA + Shrub cover + Canopy openness + Landscape structure + Humidity | 11 | 712.80 | 9.72 | 0.00 | 1.00 |
| Forest composition+Stand+Prey | Stand composition + Moth abundance:Dead tree BA + Moth abundance:Live tree BA + Moth abundance:Shrub cover + Moth abundance:Canopy Openness + Moth abundance + Dead tree BA + Live tree BA + Shrub cover + Canopy openness + Humidity | 15 | 715.99 | 12.91 | 0.00 | 1.00 |
| Forest composition | Stand composition + Humidity | 6 | 716.75 | 13.67 | 0.00 | 1.00 |
| Forest composition | Stand composition:Landscape composition + Stand composition + Landscape composition + Humidity | 8 | 717.56 | 14.48 | 0.00 | 1.00 |
| Forest composition+Stand+Prey | Stand composition + Landscape composition + Moth abundance:Dead tree BA + Moth abundance:Live tree BA + Moth abundance:Shrub cover + Moth abundance:Canopy Openness + Moth abundance + Dead tree BA + Live tree BA + Shrub cover + Canopy openness + Humidity | 16 | 718.50 | 15.42 | 0.00 | 1.00 |
| Forest composition | Stand composition + Landscape composition + Humidity | 7 | 718.98 | 15.90 | 0.00 | 1.00 |
| Stand+Prey | Moth abundance:Dead tree BA + Moth abundance:Live tree BA + Moth abundance:Shrub cover + Moth abundance:Canopy Openness + Moth abundance + Dead tree BA + Live tree BA + Shrub cover + Canopy openness + Humidity | 14 | 721.49 | 18.42 | 0.00 | 1.00 |
| Landscape | Landscape structure + Humidity | 6 | 721.56 | 18.48 | 0.00 | 1.00 |
| Forest composition | Stand composition:Landscape composition + Humidity | 6 | 722.34 | 19.26 | 0.00 | 1.00 |
| Forest composition+Stand+Prey | Landscape composition + Moth abundance:Dead tree BA + Moth abundance:Live tree BA + Moth abundance:Shrub cover + Moth abundance:Canopy Openness + Moth abundance + Dead tree BA + Live tree BA + Shrub cover + Canopy openness + Humidity | 15 | 724.34 | 21.26 | 0.00 | 1.00 |
| Prey | Moth abundance + Humidity | 6 | 727.04 | 23.96 | 0.00 | 1.00 |
| Forest composition | Landscape composition + Humidity | 6 | 727.83 | 24.75 | 0.00 | 1.00 |
| - | Null model | 4 | 735.02 | 31.94 | 0.00 | 1.00 |

1. *P. pygmaeus/M. schreibersii*

| **Model type** | **Model** | **K** | **AICc** | **ΔAICc** | **ω_i_** | **cum. ωi** |
| --- | --- | --- | --- | --- | --- | --- |
| **Forest composition+Stand+Prey** | **Stand composition:Landscape composition + Moth abundance:Shrub cover + Moth abundance + Shrub cover + Humidity** | **9** | **535.64** | **0.00** | **0.22** | **0.22** |
| **Stand** | **Shrub cover + Humidity** | **6** | **535.92** | **0.27** | **0.19** | **0.41** |
| **Forest composition+Stand+Landscape** | **Stand composition:Landscape composition + Landscape structure + Humidity** | **8** | **537.06** | **1.42** | **0.11** | **0.52** |
| **Stand+Prey** | **Moth abundance:Shrub cover + Moth abundance + Shrub cover + Humidity** | **8** | **537.62** | **1.98** | **0.08** | **0.60** |
| Forest composition | Stand composition:Landscape composition + Humidity | 6 | 538.27 | 2.62 | 0.06 | 0.66 |
| Stand+Landscape | Landscape structure + Shrub cover + Humidity | 7 | 538.31 | 2.67 | 0.06 | 0.71 |
| Forest composition+Stand+Landscape | Stand composition:Landscape composition + Stand composition + Landscape composition + Landscape structure + Humidity | 10 | 538.80 | 3.15 | 0.05 | 0.76 |
| Forest composition+Stand+Prey | Stand composition:Landscape composition + Stand composition + Landscape composition + Moth abundance:Shrub cover + Moth abundance + Shrub cover + Humidity | 11 | 539.24 | 3.60 | 0.04 | 0.79 |
| Forest composition+Stand+Prey | Landscape composition + Moth abundance:Shrub cover + Moth abundance + Shrub cover + Humidity | 9 | 539.34 | 3.70 | 0.03 | 0.83 |
| Forest composition+Stand+Landscape | Stand composition + Landscape structure + Humidity | 8 | 539.90 | 4.26 | 0.03 | 0.85 |
| Forest composition+Stand+Prey | Stand composition + Moth abundance:Shrub cover + Moth abundance + Shrub cover + Humidity | 9 | 540.10 | 4.45 | 0.02 | 0.88 |
| Forest composition+Stand+Landscape | Landscape composition + Landscape structure + Humidity | 8 | 540.20 | 4.55 | 0.02 | 0.90 |
| Prey | Moth abundance + Humidity | 6 | 540.27 | 4.63 | 0.02 | 0.92 |
| Forest composition | Stand composition + Humidity | 6 | 540.49 | 4.84 | 0.02 | 0.94 |
| Forest composition | Landscape composition + Humidity | 6 | 541.62 | 5.97 | 0.01 | 0.95 |
| Landscape | Landscape structure + Humidity | 6 | 541.68 | 6.03 | 0.01 | 0.96 |
| Forest composition+Stand+Prey | Stand composition + Landscape composition + Moth abundance:Shrub cover + Moth abundance + Shrub cover + Humidity | 10 | 541.93 | 6.29 | 0.01 | 0.97 |
| Forest composition+Stand+Landscape | Stand composition + Landscape composition + Landscape structure + Humidity | 9 | 542.01 | 6.37 | 0.01 | 0.98 |
| Forest composition | Stand composition:Landscape composition + Stand composition + Landscape composition + Humidity | 8 | 542.39 | 6.74 | 0.01 | 0.99 |
| Forest composition | Stand composition + Landscape composition + Humidity | 7 | 542.66 | 7.02 | 0.01 | 1.00 |
| - | Null model | 4 | 543.50 | 7.86 | 0.00 | 1.00 |

1. *Plecotus* spp.

| **Model type** | **Model** | **K** | **AICc** | **ΔAICc** | **ω_i_** | **cum. ωi** |
| --- | --- | --- | --- | --- | --- | --- |
| **Forest composition** | **Stand composition + Julian day** | **5** | **263.40** | **0.00** | **0.41** | **0.41** |
| **Forest composition+Stand+Prey** | **Stand composition + Moth abundance:Shrub cover + Moth abundance + Shrub cover + Julian day** | **8** | **265.12** | **1.72** | **0.18** | **0.59** |
| Forest composition | Stand composition + Landscape composition + Julian day | 6 | 265.71 | 2.31 | 0.13 | 0.72 |
| Forest composition+Stand+Landscape | Stand composition + Shrub cover + Landscape structure + Julian day | 7 | 266.89 | 3.49 | 0.07 | 0.79 |
| Forest composition+Stand+Prey | Stand composition + Landscape composition + Moth abundance:Shrub cover + Moth abundance + Shrub cover + Julian day | 9 | 267.63 | 4.22 | 0.05 | 0.84 |
| Forest composition | Stand composition:Landscape composition + Stand composition + Landscape composition + Julian day | 7 | 267.81 | 4.41 | 0.05 | 0.89 |
| Forest composition+Stand+Landscape | Stand composition + Landscape composition + Shrub cover + Landscape structure + Julian day | 8 | 269.18 | 5.78 | 0.02 | 0.91 |
| Stand+Prey | Moth abundance:Shrub cover + Moth abundance + Shrub cover + Julian day | 7 | 269.22 | 5.81 | 0.02 | 0.93 |
| Forest composition+Stand+Prey | Stand composition:Landscape composition + Stand composition + Landscape composition + Moth abundance:Shrub cover + Moth abundance + Shrub cover + Julian day | 10 | 269.36 | 5.96 | 0.02 | 0.95 |
| Forest composition+Stand+Prey | Stand composition:Landscape composition + Moth abundance:Shrub cover + Moth abundance + Shrub cover + Julian day | 8 | 269.39 | 5.98 | 0.02 | 0.98 |
| Forest composition+Stand+Landscape | Stand composition:Landscape composition + Stand composition + Landscape composition + Shrub cover + Landscape structure + Julian day | 9 | 271.27 | 7.87 | 0.01 | 0.98 |
| Forest composition+Stand+Prey | Landscape composition + Moth abundance:Shrub cover + Moth abundance + Shrub cover + Julian day | 8 | 271.59 | 8.19 | 0.01 | 0.99 |
| Prey | Moth abundance + Julian day | 5 | 272.67 | 9.26 | 0.00 | 0.99 |
| Stand | Shrub cover + Julian day | 5 | 274.22 | 10.82 | 0.00 | 1.00 |
| Stand+Landscape | Landscape structure + Shrub cover + Julian day | 6 | 275.05 | 11.64 | 0.00 | 1.00 |
| Forest composition+Stand+Landscape | Stand composition:Landscape composition + Shrub cover + Landscape structure + Julian day | 7 | 275.71 | 12.31 | 0.00 | 1.00 |
| Forest composition+Stand+Landscape | Landscape composition + Shrub cover + Landscape structure + Julian day | 7 | 276.91 | 13.51 | 0.00 | 1.00 |
| Forest composition | Stand composition:Landscape composition + Julian day | 5 | 278.19 | 14.79 | 0.00 | 1.00 |
| Landscape | Landscape structure + Julian day | 5 | 278.37 | 14.96 | 0.00 | 1.00 |
| Forest composition | Landscape composition + Julian day | 5 | 279.38 | 15.98 | 0.00 | 1.00 |
| - | Null model | 3 | 279.72 | 16.32 | 0.00 | 1.00 |

1. *R. hipposideros*

| **Model type** | **Model** | **K** | **AICc** | **ΔAICc** | **ω_i_** | **cum. ωi** |
| --- | --- | --- | --- | --- | --- | --- |
| **Stand** | **Canopy Openness + Julian day** | **6** | **361.79** | **0.00** | **0.24** | **0.24** |
| **Forest composition** | **Landscape composition + Julian day** | **6** | **363.41** | **1.62** | **0.11** | **0.35** |
| Forest composition | Stand composition:Landscape composition + Julian day | 6 | 363.86 | 2.07 | 0.09 | 0.43 |
| Stand+Landscape | Landscape structure + Canopy Openness + Julian day | 7 | 363.86 | 2.08 | 0.09 | 0.52 |
| Forest composition+Stand+Landscape | Landscape composition + Canopy Openness + Landscape structure + Julian day | 8 | 363.99 | 2.20 | 0.08 | 0.60 |
| Landscape | Landscape structure + Julian day | 6 | 365.16 | 3.37 | 0.04 | 0.64 |
| Forest composition | Stand composition + Landscape composition + Julian day | 7 | 365.30 | 3.51 | 0.04 | 0.68 |
| Forest composition+Stand+Landscape | Stand composition:Landscape composition + Canopy Openness + Landscape structure + Julian day | 8 | 365.53 | 3.74 | 0.04 | 0.72 |
| Forest composition | Stand composition + Julian day | 6 | 365.59 | 3.80 | 0.04 | 0.76 |
| Prey | Moth abundance + Julian day | 6 | 365.62 | 3.83 | 0.04 | 0.79 |
| Stand+Prey | Moth abundance:Canopy Openness + Moth abundance + Canopy Openness + Julian day | 8 | 365.72 | 3.93 | 0.03 | 0.82 |
| Forest composition+Stand+Prey | Landscape composition + Moth abundance:Canopy Openness + Moth abundance + Canopy Openness + Julian day | 9 | 365.77 | 3.98 | 0.03 | 0.86 |
| Forest composition | Stand composition:Landscape composition + Stand composition + Landscape composition + Julian day | 8 | 365.82 | 4.03 | 0.03 | 0.89 |
| Forest composition+Stand+Landscape | Stand composition + Canopy Openness + Landscape structure + Julian day | 8 | 366.10 | 4.31 | 0.03 | 0.92 |
| Forest composition+Stand+Landscape | Stand composition + Landscape composition + Canopy Openness + Landscape structure + Julian day | 9 | 366.43 | 4.64 | 0.02 | 0.94 |
| Forest composition+Stand+Prey | Stand composition:Landscape composition + Moth abundance:Canopy Openness + Moth abundance + Canopy Openness + Julian day | 9 | 367.52 | 5.74 | 0.01 | 0.95 |
| - | Null model | 4 | 367.73 | 5.94 | 0.01 | 0.97 |
| Forest composition+Stand+Landscape | Stand composition:Landscape composition + Stand composition + Landscape composition + Canopy Openness + Landscape structure + Julian day | 10 | 367.99 | 6.20 | 0.01 | 0.98 |
| Forest composition+Stand+Prey | Stand composition + Moth abundance:Canopy Openness + Moth abundance + Canopy Openness + Julian day | 9 | 368.06 | 6.28 | 0.01 | 0.99 |
| Forest composition+Stand+Prey | Stand composition + Landscape composition + Moth abundance:Canopy Openness + Moth abundance + Canopy Openness + Julian day | 10 | 368.34 | 6.56 | 0.01 | 1.00 |
| Forest composition+Stand+Prey | Stand composition:Landscape composition + Stand composition + Landscape composition + Moth abundance:Canopy Openness + Moth abundance + Canopy Openness + Julian day | 11 | 370.18 | 8.39 | 0.00 | 1.00 |

**Table S3.** Standardized, model-averaged parameter estimates with associated standards errors (SE) and 85% confidence intervals of the best GLMMs (ΔAICc <2) relating the effects of forest composition, landscape structure, stand structure, moth abundance on bat activity. Results on bat activity are presented for the 10% maximum error risk tolerance applied to the identification of bat sequences and resulting bat activity (i.e. number of 1-min intervals with ≥1 bat sequences). Variables in bold represent influential variables for which 85% CI did not overlap zero. Stand composition corresponds to the percentage cover of deciduous trees within stand while landscape composition was inferred using the proportion of deciduous forests within the forested landscape area at 1 km radius scale. Family distribution of the GLMMs is given in bracket.

| **Response variable** | **Explanatory variable** | **Estimate (±SE)** | **Lower 85** | **Upper 85** |
| --- | --- | --- | --- | --- |
| *B. barbastellus* (p) | **Stand composition:Landscape composition** | **-1.02 (±0.29)** | **-1.44** | **-0.60** |
|  | **Small-sized moth abundance:Shrub cover** | **-0.92(±0.25)** | **-1.28** | **-0.56** |
|  | **Julian day** | **0.70 (±0.21)** | **0.40** | **1.00** |
| *H. savii* (p) | Stand composition:Landscape composition | -0.02 (±0.33) | -0.50 | 0.46 |
|  | **Small-sized moth abundance:Canopy openness** | **-1.17 (±0.57)** | **-1.99** | **-0.35** |
|  | **Temperature** | **2.09 (±0.23)** | **1.76** | **2.42** |
| *M. nattereri* (p) | **Canopy openness** | **-0.41 (±0.14)** | **-0.61** | **-0.21** |
|  | **Julian day** | **0.41 (±0.21)** | **0.11** | **0.71** |
| *Nyctalus* spp. (p) | Stand composition | 0.05 (±0.13) | -0.14 | 0.24 |
|  | **Landscape composition** | **0.60 (±0.24)** | **0.25** | **0.95** |
|  | **Stand composition:Landscape composition** | **-0.18 (±0.11)** | **-0.34** | **-0.02** |
|  | Landscape structure | 0.18 (±0.22) | -0.14 | 0.50 |
|  | Medium-sized moth abundance | 0.19 (±0.19) | -0.08 | 0.46 |
|  | **Live tree basal area** | **-0.21 (±0.14)** | **-0.41** | **-0.01** |
|  | **Temperature** | **0.35 (±0.08)** | **0.23** | **0.47** |
|  | **Humidity** | **0.25 (±0.06)** | **0.16** | **0.34** |
| *P. kuhlii/nathusii* (p) | Landscape composition | -0.26 (±1.45) | -2.35 | 1.83 |
|  | Landscape structure | 0.72 (±1.25) | -1.08 | 2.52 |
|  | Small-sized moth abundance:Canopy openness | -0.41 (±2.51) | -4.02 | 3.20 |
|  | **Canopy openness** | **2.80 (±1.23)** | **1.03** | **4.57** |
|  | **Temperature** | **0.90 (±0.22)** | **0.58** | **1.22** |
| *P. pipistrellus* ^a^ (qp) | **Stand composition:Landscape composition** | **-0.43 (±0.14)** | **-0.63** | **-0.23** |
|  | **Landscape stucture** | **-0.43 (±0.26)** | **-0.80** | **-0.06** |
|  | **Live tree basal area** | **-0.66 (±0.25)** | **-1.02** | **-0.30** |
|  | Dead tree basal area | 0.26 (±0.19) | -0.01 | 0.53 |
|  | **Shrub cover** | **-1.10 (±0.23)** | **-1.43** | **-0.77** |
|  | **Canopy openness** | **-0.43 (±0.23)** | **-0.76** | **-0.10** |
|  | **Humidity** | **-0.14 (±0.04)** | **-0.20** | **-0.08** |
| *P. pygmaeus*/*M. schreibersii* ^b^ (p) | **Stand composition:Landscape composition** | **-1.27 (±0.47)** | **-1.95** | **-0.59** |
|  | **Small-sized moth abundance:Shrub cover** | **-0.72 (±0.42)** | **-1.32** | **-0.12** |
|  | **Shrub cover** | **-1.44 (±0.42)** | **-2.04** | **-0.84** |
|  | **Humidity** | **-0.19 (±0.06)** | **-0.28** | **-0.10** |
| *Plecotus* spp. (p) | **Stand composition** | **-1.68 (±0.72)** | **-2.72** | **-0.64** |
|  | Total moth abundance:Shrub cover | 0.42 (±1.10) | -1.16 | 2.00 |
|  | **Julian day** | **-1.15 (±0.41)** | **-1.74** | **-0.56** |
|  |  |  |  |  |
| *R. hipposideros* (p) | Landscape composition | 0.22 (±0.23) | -0.11 | 0.55 |
|  | **Canopy openness** | **0.38 (±0.22)** | **0.06** | **0.70** |
|  | **Julian day** | **0.57 (±0.24)** | **0.22** | **0.92** |

^a^ Only recordings from the Batlogger were considered, ^b^ One outlier was removed.

Distribution family: p Poisson, qp quasi-Poisson.

**Table S4.** Results of the spotlight analysis conducted to explore (i) the interactive effects of forest composition at both stand and landscape scales on bat activity; and (ii) the interactive effect between shrub cover and moth abundance on *Barbastella barbastellus* activity. Estimates with associated standards errors (SE) and lower and upper 95% confidence intervals (CI) are given for each explanatory variable at each level of moderation (i.e. mean ± one SD of the moderating variable).

| **Response variable** | **Explanatory variable \| Moderating variable** | **Estimate (±SE)** | **Lower 95** | **Upper 95** |
| --- | --- | --- | --- | --- |
|  | **Stand composition \| Landscape composition** |  |  |  |
| *B. barbastellus* | 51% of deciduous forests | -0.752 (±0.189) | -1.128 | -0.376 |
|  | 35% of deciduous forests | 0.000 (±0.000) | 0.000 | 0.000 |
|  | 19% of deciduous forests | 0.752 (±0.189) | 0.376 | 1.128 |
| *P. pipistrellus ^a^* | 51% of deciduous forests | -0.427 (±0.145) | -0.716 | -0.138 |
|  | 35% of deciduous forests | 0.000 (±0.000) | 0.000 | 0.000 |
|  | 19% of deciduous forests | 0.427 (±0.145) | 0.138 | 0.716 |
| *P. pygmaeus/M. schreibersii ^b^* | 51% of deciduous forests | -0.313 (±0.157) | -0.626 | <0.000 |
|  | 35% of deciduous forests | 0.000 (±0.000) | 0.000 | 0.000 |
|  | 19% of deciduous forests | 0.313 (±0.157) | >0.000 | 0.626 |
| *Nyctalus* spp. | 51% of deciduous forests | -0.024 (±0.136) | -0.295 | 0.247 |
|  | 35% of deciduous forests | 0.144 (±0.115) | -0.085 | 0.372 |
|  | 19% of deciduous forests | 0.311 (±0.168) | -0.023 | 0.645 |
|  | **Moth abundance \| Shrub cover** |  |  |  |
| *B. barbastellus* | 100% of shrub cover | -0.960 (±0.293) | -1.543 | -0.377 |
|  | 73% of shrub cover | -0.167 (±0.189) | -0.543 | 0.209 |
|  | 46% of shrub cover | 0.626 (±0.256) | 0.116 | 1.136 |

^a^ Only recordings from the Batlogger were considered, ^b^ One outlier was removed.

**Table S5**. Summary table of the amount of forest cover and proportion of deciduous forest within the forested landscape around the mixed/deciduous-dominated stands and coniferous-dominated stands and across spatial scales.

| **Variable** | **Mixed and deciduous-dominated stands Mean (± SD)** | **Coniferous-dominated stands Mean (± SD)** |
| --- | --- | --- |
| Forest cover at 1 km radius scale (%) | 81.58 (± 11.21) | 80.55 (± 11.20) |
| Forest cover at 2 km radius scale (%) | 73.07 (± 11.92) | 73.80 (± 11.81) |
| Forest cover at 3 km radius scale (%) | 68.65 (± 9.26) | 68.80 (± 9.12) |
| Proportion of deciduous forest within the forested landscape at 1 km radius scale (%) | 37.02 (± 16.31) | 33.50 (± 15.89) |
| Proportion of deciduous forest within the forested landscape at 2 km radius scale (%) | 37.90 (± 14.29) | 37.43 (± 13.47) |
| Proportion of deciduous forest within the forested landscape at 3 km radius scale (%) | 39.27 (± 11.68) | 39.04 (± 11.69) |

**Table S6.** Information on the diet and morphology of the main bat species recorded over sites.

| **Species** | **% of Lepidoptera in diet^1-4^** | **Body mass (g)^5^** | **C-M3 length (mm)^5^** | **Small prey** | **Reference on diet** |
| --- | --- | --- | --- | --- | --- |
| *Barbastella barbastellus* | 78 | 7-10 | 4.5-4.8 |  | Andreas, et al. ^6^ |
| *Myotis nattereri* | 14 | 7-10 | 5.6-6.3 |  | Swift and Racey ^7^ |
| *Hypsugo savii* | 6 | 5-9 | 4.4-4.9 | X | Dietz, et al. ^5^ |
| *Miniopterus schreibersii* | 75 | 10-14 | 5.3-6.2 |  | Aizpurua, et al. ^3^ |
| *Nyctalus noctula* | 27 | 21-30 | 7.0-8.3 |  | Jones ^8^ |
| *N. leisleri* | 21 | 13-18 | 5.3-6.1 |  | Kaňuch, et al. ^9^ |
| *Pipistrellus kuhlii* | 16 | 5-8 | 4.7-5.1 | X | Cohen, et al. ^10^ |
| *P. nathusii* | 7 | 6-10 | 4.5-4.8 | X | Krüger, et al. ^11^ |
| *P. pipistrellus* | 18 | 3-7 | 3.8-4.4 | X | Barlow ^12^ |
| *P. pygmaeus* | 5 | 4-7 | 3.7-4.4 | X | Barlow ^12^ |
| *Plecotus auritus* | 69 | 6-9 | 5.0-5.8 |  | Andriollo, et al. ^13^ |
| *P. austriacus* | 75 | 6-10 | 5.4-6.5 |  | Andriollo, et al. ^14^ |
| *Rhinolophus hipposideros* | 31 | 4-7 | 5.0-5.5 | X | Baroja, et al. ^15^ |

C-M3 length = upper tooth row length (canine – 3^rd^ molar)

Prey size was inferred based on bat morphology (body mass and upper tooth row length) and main prey items targeted.

References

1 Ware, R. L., Garrod, B., Macdonald, H. & Allaby, R. G. Guano morphology has the potential to inform conservation strategies in British bats. *PloS ONE* **15**, e0230865 (2020).

2 Goiti, U., Vecin, P., Garin, I., Saloña, M. & Aihartza, J. R. Diet and prey selection in Kuhl’s pipistrelle Pipistrellus kuhlii (Chiroptera: Vespertilionidae) in south-western Europe. *Acta Theriologica* **48**, 457-468 (2003).

3 Aizpurua, O. *et al.* Agriculture shapes the trophic niche of a bat preying on multiple pest arthropods across Europe: Evidence from DNA metabarcoding. *Molecular ecology* **27**, 815-825 (2018).

4 Kipson, M. *et al.* Foraging habitat, home-range size and diet of a Mediterranean bat species, Savi's pipistrelle. *Acta Chiropterologica* **20**, 351-360 (2018).

5 Dietz, C., Nill, D. & von Helversen, O. *Bats of Britain, Europe and Northwest Africa*. (A & C Black, 2009).

6 Andreas, M., Reiter, A. & Benda, P. Prey selection and seasonal diet changes in the western barbastelle bat (*Barbastella barbastellus*). *Acta Chiropterologica* **14**, 81-92 (2012).

7 Swift, S. & Racey, P. Gleaning as a foraging strategy in Natterer's bat *Myotis nattereri*. *Behavioral Ecology and Sociobiology* **52**, 408-416 (2002).

8 Jones, G. Flight performance, echolocation and foraging behaviour in noctule bats *Nyctalus noctula*. *Journal of Zoology* **237**, 303-312 (1995).

9 Kaňuch, P., Krištín, A. & Krištofík, J. Phenology, diet, and ectoparasites of Leisler's bat (*Nyctalus leisleri*) in the Western Carpathians (Slovakia). *Acta Chiropterologica* **7**, 249-257 (2005).

10 Cohen, Y., Bar‐David, S., Nielsen, M., Bohmann, K. & Korine, C. An appetite for pests: Synanthropic insectivorous bats exploit cotton pest irruptions and consume various deleterious arthropods. *Molecular ecology* **29**, 1185-1198 (2020).

11 Krüger, F., Clare, E. L., Symondson, W. O., Keišs, O. & Pētersons, G. Diet of the insectivorous bat *Pipistrellus nathusii* during autumn migration and summer residence. *Molecular Ecology* **23**, 3672-3683 (2014).

12 Barlow, K. E. The diets of two phonic types of the bat *Pipistrellus pipistrellus* in Britain. *Journal of Zoology* **243**, 597-609 (1997).

13 Andriollo, T., Gillet, F., Michaux, J. R. & Ruedi, M. The menu varies with metabarcoding practices: A case study with the bat *Plecotus auritus*. *PloS one* **14**, e0219135 (2019).

14 Andriollo, T., Michaux, J. & Ruedi, M. Food for everyone: differential feeding habits of cryptic bat species inferred from DNA metabarcoding. *Authorea Preprints* (2020).

15 Baroja, U. *et al.* Pest consumption in a vineyard system by the lesser horseshoe bat (*Rhinolophus hipposideros*). *PloS one* **14**, e0219265 (2019).

**Table S7.** Description of the GLMMs relating the effects of stand structure on bat activity. The null model is also displayed for comparison. Results on bat activity are presented for the 50% maximum error risk tolerance applied to the identification of bat sequences and resulting bat activity (i.e. number of 1-min intervals with ≥1 bat sequences). Models are ranked in ascending order of AICc and the number of parameters (K), AICc weight (ωi), cumulative weight (cum. ωi) are given for each model.

| **Taxa** | Model | **K** | **AICc** | **ΔAICc** | **ω_i_** | **cum. ωi** |
| --- | --- | --- | --- | --- | --- | --- |
| *B. barbastellus* | Shrub cover + Julian day | 6 | 466.00 | 0.00 | 0.93 | 0.93 |
|  | Dead tree BA + Live tree BA + Shrub cover + Canopy openness + Julian day | 9 | 472.94 | 6.94 | 0.03 | 0.96 |
|  | Canopy openness + Julian day | 6 | 473.76 | 7.77 | 0.02 | 0.98 |
|  | Dead tree BA + Julian day | 6 | 474.45 | 8.45 | 0.01 | 0.99 |
|  | Live tree BA + Julian day | 6 | 475.02 | 9.02 | 0.01 | 1.00 |
|  | Null model | 4 | 477.60 | 11.60 | 0.00 | 1.00 |
| *H. savii* | Canopy openness + Temperature | 6 | 358.29 | 0.00 | 0.79 | 0.79 |
|  | Dead tree BA + Live tree BA + Shrub cover + Canopy openness + Temperature | 9 | 361.02 | 2.73 | 0.20 | 0.99 |
|  | Shrub cover + Temperature | 6 | 368.37 | 10.08 | 0.01 | 1.00 |
|  | Dead tree BA + Temperature | 6 | 370.55 | 12.26 | 0.00 | 1.00 |
|  | Live tree BA + Temperature | 6 | 371.10 | 12.81 | 0.00 | 1.00 |
|  | Null model | 4 | 372.31 | 14.02 | 0.00 | 1.00 |
| *M. nattereri* | Canopy openness + Julian day | 5 | 327.13 | 0.00 | 0.68 | 0.68 |
|  | Dead tree BA + Julian day | 5 | 331.01 | 3.89 | 0.10 | 0.78 |
|  | Shrub cover + Julian day | 5 | 331.70 | 4.58 | 0.07 | 0.85 |
|  | Live tree BA + Julian day | 5 | 331.90 | 4.78 | 0.06 | 0.92 |
|  | Null model | 3 | 332.39 | 5.26 | 0.05 | 0.96 |
|  | Dead tree BA + Live tree BA + Shrub cover + Canopy openness + Julian day | 8 | 333.05 | 5.92 | 0.04 | 1.00 |
| *Nyctalus* spp. | Live tree BA + Temperature + Humidity | 6 | 621.16 | 0.00 | 0.41 | 0.41 |
|  | Shrub cover + Temperature + Humidity | 6 | 621.50 | 0.34 | 0.34 | 0.75 |
|  | Canopy openness + Temperature + Humidity | 6 | 623.50 | 2.34 | 0.13 | 0.88 |
|  | Dead tree BA + Temperature + Humidity | 6 | 624.01 | 2.85 | 0.10 | 0.97 |
|  | Dead tree BA + Live tree BA + Shrub cover + Canopy openness + Temperature + Humidity | 9 | 626.58 | 5.42 | 0.03 | 1.00 |
|  | Null model | 3 | 650.21 | 29.05 | 0.00 | 1.00 |
| *P. kuhlii/nathusii* | Dead tree BA + Live tree BA + Shrub cover + Canopy openness + Temperature | 9 | 382.22 | 0.00 | 0.57 | 0.57 |
|  | Canopy openness + Temperature | 6 | 382.79 | 0.57 | 0.43 | 0.99 |
|  | Dead tree BA + Temperature | 6 | 392.88 | 10.66 | 0.00 | 1.00 |
|  | Live tree BA + Temperature | 6 | 393.49 | 11.27 | 0.00 | 1.00 |
|  | Null model | 4 | 396.02 | 13.79 | 0.00 | 1.00 |
|  | Shrub cover + Temperature | 6 | 396.53 | 14.31 | 0.00 | 1.00 |
| *P. pipistrellus* ^a^ | Dead tree BA + Live tree BA + Shrub cover + Canopy openness + Humidity | 9 | 710.43 | 0.00 | 0.81 | 0.81 |
|  | Shrub cover + Humidity | 6 | 713.37 | 2.94 | 0.19 | 0.99 |
|  | Dead tree BA + Humidity | 6 | 720.32 | 9.88 | 0.01 | 1.00 |
|  | Canopy openness + Humidity | 6 | 723.06 | 12.63 | 0.00 | 1.00 |
|  | Live tree BA + Humidity | 6 | 727.68 | 17.25 | 0.00 | 1.00 |
|  | Null model | 4 | 735.02 | 24.59 | 0.00 | 1.00 |
| *P. pygmaeus*/*M. schreibersii ^b^* | Shrub cover + Humidity | 6 | 535.92 | 0.00 | 0.58 | 0.58 |
|  | Canopy openness + Humidity | 6 | 537.53 | 1.61 | 0.26 | 0.83 |
|  | Dead tree BA + Humidity | 6 | 540.16 | 4.24 | 0.07 | 0.90 |
|  | Dead tree BA + Live tree BA + Shrub cover + Canopy openness + Humidity | 9 | 540.85 | 4.94 | 0.05 | 0.95 |
|  | Live tree BA + Humidity | 6 | 541.50 | 5.59 | 0.04 | 0.99 |
|  | Null model | 4 | 543.50 | 7.58 | 0.01 | 1.00 |
| *Plecotus* spp. | Shrub cover + Julian day | 5 | 274.22 | 0.00 | 0.58 | 0.58 |
|  | Canopy openness + Julian day | 5 | 276.26 | 2.04 | 0.21 | 0.79 |
|  | Live tree BA + Julian day | 5 | 278.38 | 4.15 | 0.07 | 0.86 |
|  | Dead tree BA + Live tree BA + Shrub cover + Canopy openness + Julian day | 8 | 278.65 | 4.43 | 0.06 | 0.92 |
|  | Dead tree BA + Julian day | 5 | 279.64 | 5.42 | 0.04 | 0.96 |
|  | Null model | 3 | 279.72 | 5.50 | 0.04 | 1.00 |
| *R. hipposideros* | Canopy openness + Julian day | 6 | 361.79 | 0.00 | 0.45 | 0.45 |
|  | Dead tree BA + Live tree BA + Shrub cover + Canopy openness + Julian day | 9 | 362.65 | 0.86 | 0.30 | 0.75 |
|  | Dead tree BA + Julian day | 6 | 365.16 | 3.37 | 0.08 | 0.83 |
|  | Shrub cover + Julian day | 6 | 365.19 | 3.40 | 0.08 | 0.92 |
|  | Live tree BA + Julian day | 6 | 365.82 | 4.03 | 0.06 | 0.98 |
|  | Null model | 4 | 367.73 | 5.94 | 0.02 | 1.00 |

^a^ Only recordings from the Batlogger were considered, ^b^ One outlier was removed.

BA: basal area.

**Table S8.** Description of the GLMMs relating the effects of moth abundance on bat activity. The null model is also displayed for comparison. Results on bat activity are presented for the 50% maximum error risk tolerance applied to the identification of bat sequences and resulting bat activity (i.e. number of 1-min intervals with ≥1 bat sequences). Models are ranked in ascending order of AICc and the number of parameters (K), AICc weight (ωi), cumulative weight (cum. ωi) are given for each model.

| **Taxa** | **Model** | **K** | **AICc** | **ΔAICc** | **ω_i_** | **cum. ωi** |
| --- | --- | --- | --- | --- | --- | --- |
| *B. barbastellus* | Abundance of small-sized moths + Julian day | 6 | 475.31 | 0.00 | 0.24 | 0.24 |
|  | Total moth abundance + Julian day | 6 | 475.43 | 0.12 | 0.23 | 0.47 |
|  | Abundance of medium-sized moths + Julian day | 6 | 475.45 | 0.14 | 0.23 | 0.70 |
|  | Abundance of large-sized moths + Julian day | 6 | 475.45 | 0.14 | 0.23 | 0.92 |
|  | Null model | 4 | 477.60 | 2.29 | 0.08 | 1.00 |
| *M. nattereri* | Abundance of small-sized moths + Julian day | 5 | 330.59 | 0.00 | 0.31 | 0.31 |
|  | Abundance of medium-sized moths + Julian day | 5 | 331.04 | 0.45 | 0.24 | 0.55 |
|  | Abundance of large-sized moths + Julian day | 5 | 331.78 | 1.19 | 0.17 | 0.72 |
|  | Total moth abundance + Julian day | 5 | 331.92 | 1.33 | 0.16 | 0.88 |
|  | Null model | 3 | 332.39 | 1.80 | 0.12 | 1.00 |
| *Nyctalus* spp. | Abundance of medium-sized moths + Temperature + Humidity | 6 | 622.01 | 0.00 | 0.35 | 0.35 |
|  | Total moth abundance + Temperature + Humidity | 6 | 622.68 | 0.67 | 0.25 | 0.60 |
|  | Abundance of large-sized moths + Temperature + Humidity | 6 | 622.77 | 0.77 | 0.24 | 0.84 |
|  | Abundance of small-sized moths + Temperature + Humidity | 6 | 623.63 | 1.62 | 0.16 | 1.00 |
|  | Null model | 3 | 650.21 | 28.20 | 0.00 | 1.00 |
| *Plecotus* spp. | Total moth abundance + Julian day | 5 | 272.67 | 0.00 | 0.49 | 0.49 |
|  | Abundance of medium-sized moths + Julian day | 5 | 274.44 | 1.78 | 0.20 | 0.69 |
|  | Abundance of large-sized moths + Julian day | 5 | 274.96 | 2.29 | 0.15 | 0.84 |
|  | Abundance of small-sized moths + Julian day | 5 | 275.08 | 2.42 | 0.15 | 0.99 |
|  | Null model | 3 | 279.72 | 7.05 | 0.01 | 1.00 |

**Table S9.** Results from the Principal component analyses (PCA) of variables describing the forest at the landscape scale. Landscape variables were calculated at 1 km radius buffer around the sampling points. Only the first PCA axis was retained for inclusion in the models.

| **Landscape variables** | **PC1** | **PC2** | **PC3** | **PC4** |
| --- | --- | --- | --- | --- |
| Forest cover | 0.47 | -0.67 | -0.55 | -0.14 |
| Forest edge density | -0.46 | -0.73 | 0.50 | 0.00 |
| Mean forest patch area | 0.54 | -0.09 | 0.38 | 0.75 |
| No. of forest patches | -0.53 | -0.04 | -0.55 | 0.65 |
|  |  |  |  |  |
| Variation explained (%) | 0.85 | 0.11 | 0.05 | <0.01 |
| Eigenvalue | 3.39 | 0.42 | 0.19 | <0.01 |


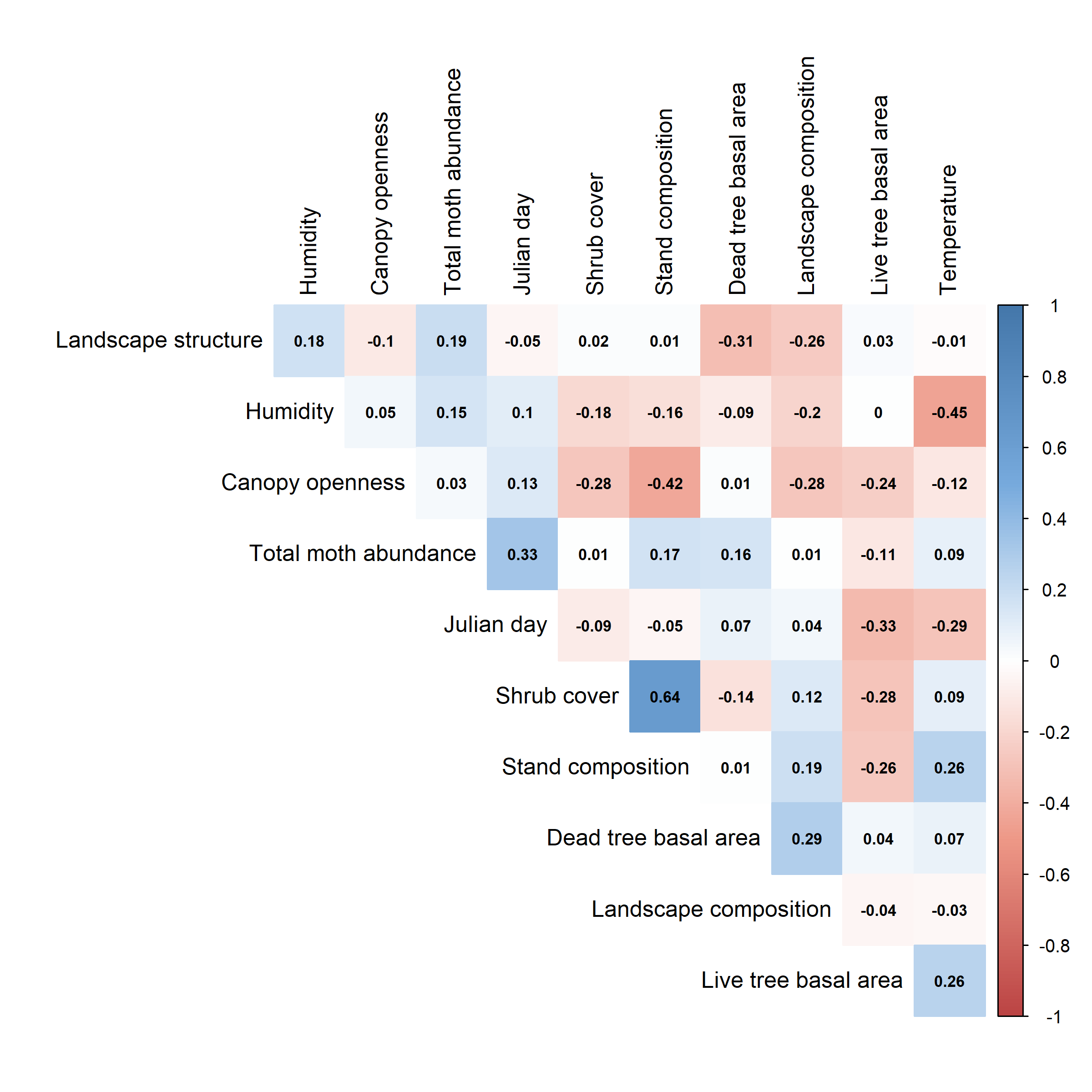


**Fig. S1** Correlation matrix between the explanatory variables. Values represent the Spearman’s correlation coefficient. Stand composition corresponds to the percentage of deciduous trees within stand and landscape composition to the percentage of deciduous forests within the forested landscape at 1 km radius scale around the sampling sites.
